# Pulmonary Disease and NF-κB-Associated Responses in Mice Infected with Two *Klebsiella pneumoniae* Isolates

**DOI:** 10.3390/microorganisms14102337

**Published:** 2026-10-13

**Authors:** Rongrong Song, Jiuyang Xu, Danni Pu, Na Wang, Muhammad Ali Syed, Jiankang Zhao, Bin Cao

**Affiliations:** 1 Changping Laboratory, Chinese Academy of Medical Sciences & Peking Union Medical College, Beijing 102206, China; 2 Department of Pulmonary and Critical Care Medicine, Center of Respiratory Medicine, China-Japan Friendship Hospital, National Center for Respiratory Medicine, State Key Laboratory of Respiratory Health and Multimorbidity, National Clinical Research Center for Respiratory Diseases, Institute of Respiratory Medicine, Chinese Academy of Medical Sciences, Beijing 100029, China; 3 China-Japan Friendship Hospital (Institute of Clinical Medical Sciences), Chinese Academy of Medical Sciences & Peking Union Medical College, Beijing 100029, China; 4 Department of Microbiology, The University of Haripur, Haripur 22620, Pakistan

**Keywords:** bacterial pneumonia, hypervirulent *Klebsiella pneumoniae*, single-cell RNA sequencing, macrophages, NF-κB

## Abstract

Hypervirulent *Klebsiella pneumoniae* (hvKP) causes invasive disease, but pulmonary macrophage responses remain incompletely defined. We compared strain 55055, used as a classical *K. pneumoniae* (cKP) comparator, with strain 46274, operationally classified as hvKP, using a pneumonia model, single-cell RNA sequencing of pooled lungs, and RAW 264.7 macrophages. At the same inoculum, strain 46274 had higher lung burdens and greater mortality than strain 55055; representative sections showed more extensive inflammatory-cell infiltration, whereas no pairwise difference in animal-level H&E scores remained significant after multiple-comparison adjustment. Because neither pulmonary burden nor disease stage was matched, immune-response differences cannot be separated from effects of unequal bacterial exposure or differences in disease progression. Descriptive pooled-library analysis showed differences in immune-cell composition and CellChat-inferred communication. In the single day-2 strain 46274 pooled library, SPP1^+^ macrophages comprised a larger fraction than in the corresponding strain 55055 pooled library and had high inflammatory-response, chemokine, and bacterial-recognition gene-set scores; this library-level observation was not independently validated in individual animals. Tissue-level staining showed numerical day-2 differences in CD86- and CD206-positive-cell abundance, but adjusted pairwise comparisons did not confirm strain-specific differences and the data do not demonstrate M1/M2 macrophage polarization. NF-κB-associated imaging findings remained qualitative. In vitro, strain 46274 showed lower adhesion to and phagocytosis by RAW 264.7 macrophages, but higher IL-6 and TNF-α readouts and stronger qualitative late p65 nuclear localization. These in vitro observations were obtained in RAW 264.7 cells within one experimental run and should not be extrapolated directly to primary pulmonary macrophages. Overall, in this strain-pair murine model, strain 46274 was associated with more severe pulmonary disease and NF-κB-associated inflammatory readouts.

## 1. Introduction

*Klebsiella pneumoniae* (KP) is an encapsulated Gram-negative opportunistic pathogen that colonizes the gastrointestinal and upper respiratory tracts [1,2]. Classical *K. pneumoniae* (cKP) mainly affects neonates, older adults, and immunocompromised individuals, whereas hypervirulent *K. pneumoniae* (hvKP) can infect healthy community-dwelling individuals and cause tissue invasion, liver abscess, and hematogenous dissemination [2,3,4]. Major virulence determinants include capsule, lipopolysaccharide, fimbriae, and siderophores, which support immune evasion, adhesion, colonization, and iron acquisition [1,2,4]. *K. pneumoniae* readily acquires and disseminates antimicrobial-resistance determinants [5]. In July 2024, the World Health Organization (WHO) reported the global emergence of carbapenemase-producing hvKP ST23: 16 of 43 responding countries, territories, and areas reported hvKP, 12 reported ST23-K1, and detections spanned all six WHO regions [6]. WHO warned that convergence of hypervirulence and carbapenem resistance can cause severe invasive infections in healthy and immunocompromised people while restricting treatment options [6]. Although global hvKP-specific mortality is unknown, *K. pneumoniae* was associated with an estimated 790,000 deaths (95% uncertainty interval, 571,000–1,060,000) worldwide in 2019 [7].

The hvKP capsule reduces macrophage uptake [8], and hvKP can replicate within hepatic Kupffer cells, whereas Kupffer-cell killing limits hepatic accumulation [9,10]. RmpA/RmpA2-mediated hypermucoviscosity impairs neutrophil phagocytosis [11], while ST23/ST86, K1/K2, and hypermucoviscous isolates show complement-, serum-, and phagocyte-evasion phenotypes in strain- and model-specific studies [12,13,14]. Wzi and RfaH promote capsule-associated persistence, dissemination, and pulmonary injury [15,16]. Conversely, K1-capsule antibodies, Dep1979, and isoferulic acid enhance macrophage uptake or host clearance [17,18,19]. Because macrophages coordinate phagocytosis, inflammatory-mediator release, and antigen presentation [20], disruption of these functions may contribute to severe hvKP disease. Macrophage inflammatory signaling, cell death, and metabolism vary across hvKP isolates and models. Hcp-associated HMGB1 release was linked to endothelial pyroptosis [21], and STAT1-dependent alveolar-macrophage PANoptosis to lung injury [22]. Other studies implicated NF-κB signaling [23], exosomal miR-155-5p [24], and mTOR-dependent lipid droplets [25]. Isolate-specific work also reported lower cytokine responses with reduced uptake [26], HDAC2-linked autophagy repression and an M2-like state [27], or late SLC7A11/GSH activation with reduced TLR4 signaling [28].

SPP1 (osteopontin) has been linked to pulmonary host responses. In murine *K. pneumoniae* pneumonia, global osteopontin deficiency impaired early neutrophil recruitment and increased subsequent lung bacterial burdens and mortality [29]; this was not a macrophage-specific manipulation. In experimental SARS-CoV-2 pneumonia, single-cell and spatial analyses identified monocyte-derived Slamf9^+^ macrophages expressing Spp1 that accumulated during infection and were associated with viral clearance and later inflammation resolution [30]. The abundance and transcriptional features of SPP1-expressing macrophages in hvKP pneumonia remain poorly defined.

Against this background, we compared clinical isolates 55055 and 46274 using a murine pneumonia model, pooled-lung single-cell RNA sequencing, and RAW 264.7 cells. We assessed disease severity and pulmonary immune-cell composition, with emphasis on SPP1^+^ macrophages, and examined adhesion, phagocytosis, inflammatory-mediator production, and NF-κB-associated readouts. Throughout the manuscript, cKP and hvKP are used as operational labels for this strain pair rather than as claims that either isolate represents its broader clinical category. The operational designation of 46274 was based on the previously documented lethality phenotype, string test, and genomic background, rather than on any single definitive marker [31].

## 2. Materials and Methods

### 2.1. Cell Culture

Murine RAW 264.7 macrophages (Procell, Wuhan, China) were maintained in DMEM with 10% fetal bovine serum (Gibco/Thermo Fisher, Scoresby, Australia, 10100147) at 37 °C and 5% CO_2_ and authenticated by STR profiling.

### 2.2. Bacterial Strains and Characterization

Two previously archived clinical *K. pneumoniae* isolates from bronchoalveolar lavage fluid were cultured on blood agar at 37 °C. Previously generated whole-genome data identified 55055 as ST11, KL64/K64, O1/O2v1 (predicted O2a), with ybt9/iuc1 and blaKPC-2/blaCTX-M-65; 46274 was ST65-1LV, KL2/K2, O1/O2v2 (predicted O1), with ybt17/clb3/iuc1/iro1, a truncated rmp1/KpVP-1-associated locus, and blaCTX-M-15. Because no single phenotypic or genomic marker is definitive [31], 46274 was operationally classified as hvKP based on a prior high-lethality murine intraperitoneal challenge, supported by this genomic background and a positive string test; string-test-negative 55055 served as the cKP comparator. This operational comparison should not be interpreted as a general test of all hvKP and cKP lineages.

### 2.3. Construction of sfGFP-Labeled Strains

Forhigh Biotech (Hangzhou, China) generated sfGFP-expressing derivatives by electroporating the 5855 bp pY12Cm-sfgfp plasmid at 2500 V and selecting with 34 μg/mL chloramphenicol. Strains were passaged with 34 μg/mL chloramphenicol, and designated cKP-sfGFP and hvKP-sfGFP. Before infection, the sfGFP derivatives and their corresponding parental strains were adjusted to the same McFarland turbidity and used under the same infection conditions. No parental-versus-sfGFP growth-curve or inoculum CFU-equivalence experiment was performed; these operational controls do not establish functional equivalence.

### 2.4. Mouse Pulmonary Infection Model

Female 6–8-week-old C57BL/6 mice were obtained from Beijing SPF Biotechnology (Beijing, China). Under intraperitoneal sodium pentobarbital anesthesia, mice in the infected groups received an intranasal inoculation of 6.5 × 10^6^ CFU/mL of strain 55055 or 46274 in 30 μL; experiments that included a PBS control used 30 μL PBS. The final survival/body-weight experiment was a separate cohort comprising four mice per infected group (*n* = 4/strain) and did not include a contemporaneous PBS group. A separate terminal-tissue cohort comprised PBS mice sampled on day 4, 55055-infected mice sampled on days 1, 2, and 4, and 46274-infected mice sampled on days 1 and 2 (*n* = 3 mice per condition, with no exclusions). H&E, immunohistochemistry, lung p65 immunofluorescence, and pooled-lung single-cell RNA sequencing were performed using this terminal-tissue cohort. Lung bacterial-burden measurements and day-1 Western blotting were performed in a separate cohort; three mice per infection group supplied the day-1 Western-blot samples. Body weight and survival were monitored for 7 days. The procedures were approved by the Institutional Animal Care and Use Committee of the Institute of Biophysics, Chinese Academy of Sciences (ABSL-2-2025073; 31 July 2025).

### 2.5. Quantification of Bacterial Burden in Lung Tissues

Lungs were weighed, homogenized in sterile PBS, serially diluted, and plated on LB agar overnight at 37 °C; burden was reported as CFU·mg^−1^ of lung tissue. For the plotted burden data, sample sizes were 55055 *n* = 4 and 46274 *n* = 5 on day 1, 55055 *n* = 6 and 46274 *n* = 6 on day 2. Survival and terminal tissue collection were separate endpoints. The bacterial-burden cohort was separate from the terminal-tissue cohort used for H&E, immunohistochemistry, lung p65 immunofluorescence, and pooled-lung single-cell RNA sequencing; day-1 lungs from the burden cohort also supplied the Western-blot samples.

### 2.6. Histopathological Staining of Lung Tissues

Left lungs were fixed in 4% paraformaldehyde, paraffin-embedded, sectioned at 4 μm, and H&E-stained. This study provided one overall lung-injury severity score for each mouse-linked sample (PBS, day 4; 55055, days 1, 2, and 4; 46274, days 1 and 2; *n* = 3 mice per condition). This assessment section cited INHAND terminology and defined an ordinal severity scale: 0, normal; 1, minimal; 2, mild; 3, moderate; and 4, severe. This study did not identify the individual assessor or document blinding or the number and selection of microscopic fields used for scoring. Each mouse was treated as the experimental unit. Ordinal scores were summarized as medians with ranges in the text; individual mouse scores and mean ± SD are shown descriptively. The six conditions were compared using a tie-corrected Kruskal–Wallis test (chi-square approximation, df = 5), followed by Dunn’s test across all 15 pairwise comparisons. This exploratory analysis compared the observed conditions without estimating separate strain, time, or interaction effects.

### 2.7. Immunohistochemical Staining

Paraffin lung sections underwent antigen retrieval and blocking before staining with CD206 (Cell Signaling Technology, Danvers, MA, USA, 24595), CD86 (Cell Signaling Technology, Danvers, MA, USA, 19589), or Ly6G (Abcam, Cambridge, UK, ab238132) primary antibodies. Signals were developed with appropriate secondary antibodies and 3,3′-diaminobenzidine, counterstained with hematoxylin, and examined by light microscopy. CD206, CD86, and Ly6G staining was quantified with Aipathwell^®^ AI image-analysis software. Positive-cell proportion was expressed as the percentage of positive cells among all cells. Mouse-level marker proportions were recorded for *n* = 3 mice per group at each available time point; thisstudy did not document the number or selection of microscopic fields or blinding. The six conditions (PBS on day 4, strain 55055 on days 1, 2, and 4, and strain 46274 on days 1 and 2) were analyzed separately for each marker using a tie-corrected Kruskal–Wallis test (chi-square approximation, df = 5), followed by Dunn’s test for all 15 pairwise comparisons within that marker. Individual mouse values and mean ± SD are reported descriptively. No macrophage-marker co-localization was performed; these measurements therefore represent tissue-level marker abundance and do not establish M1/M2 macrophage proportions.

### 2.8. Cell-Associated sfGFP Imaging and p65 Staining

Cell-associated cKP-sfGFP or hvKP-sfGFP signals in RAW 264.7 cells (MOI 50) were assessed by fluorescence microscopy. Cells were fixed, permeabilized, and counterstained with DAPI, and whole-slide images were quantified in Fiji (ImageJ v1.54p) as relative sfGFP intensity (sfGFP/DAPI). NF-κB p65 localization in RAW 264.7 cells challenged at MOI 50 and in lung sections was assessed with kit SN368 (Beyotime, Shanghai, China). Lung assessment included one section and one representative field from each of three mice/group. The microscopy data table contained 6 control observations and 55055/46274 observation counts of 11/6, 11/12, and 10/6 at 3, 6, and 9 h, respectively. The displayed in vitro microscopy observations were generated in one experimental run, and the recorded wells or image fields do not represent independent experimental repeats; the microscopy results are therefore interpreted descriptively.

### 2.9. Enzyme-Linked Immunosorbent Assay (ELISA)

RAW 264.7 cells were challenged with 55055 or 46274 (MOI 1). At 24 h, supernatant IL-6 and TNF-α were measured with ELISA kits EK206 and EK282, respectively (MultiSciences Biotech, Hangzhou, China). Absorbance was read at 450 and 570 nm; background-subtracted concentrations were calculated from standard curves. The TNF-α ELISA used *n* = 4 recorded observations per group; the IL-6 ELISA used *n* = 3 for the uninfected control and *n* = 4 for each bacterial-strain group. MOI 50 (and MOI 100 where included for CFU inoculum-dependence comparisons) was selected on the basis of preliminary condition-setting experiments for early RAW 264.7 uptake/phagocytosis readouts. The rationale for the subsequent MOI settings was the same. MOI 1 was used for 24 h cytokine and transcript sampling to reduce premature cell death before collection.

### 2.10. Flow-Cytometric Cell-Associated sfGFP Assay

Cell-associated cKP-sfGFP or hvKP-sfGFP signals in RAW 264.7 cells (MOI 50) were analyzed at 0, 3, 6, and 9 h in the FITC channel of a CytoFLEX flow cytometer (Beckman Coulter, Brea, CA, USA). The recorded replicate numbers were *n* = 6 for the uninfected control, *n* = 6/6 for 55055/46274 at 3 h, *n* = 6/5 at 6 h, and *n* = 6/6 at 9 h. Uninfected cells defined the sfGFP-positive threshold, and the percentage of sfGFP-positive cells was recorded as the cell-associated sfGFP-positive fraction; data were analyzed in FlowJo v10.9.0. This fluorescence readout does not by itself distinguish internalized from surface-associated bacteria. The displayed flow-cytometry data were generated in one experimental run using within-run biological wells and do not represent independent experimental repeats.

### 2.11. Western Blotting

Lung proteins from three independently sampled mice in each of the PBS, 55055, and 46274 groups were extracted separately in inhibitor-containing lysis buffer (Thermo Fisher, Waltham, MA, USA, 78442); infected lungs were collected on day 1. Proteins were quantified by BCA assay, separated by SDS-PAGE, and transferred to PVDF. Proteintech (Wuhan, China) antibodies targeted phospho-NF-κB p65 (82335-1-RR), NF-κB p65 (80979-1-RR), HRP-conjugated β-actin (60008), HRP-conjugated anti-rabbit (RGAR001). For the quantification, phospho-p65 band intensity was normalized to the corresponding β-actin band intensity, yielding one p-p65/β-actin value per mouse. Each mouse was treated as an independent biological replicate (*n* = 3 per group). Original uncropped immunoblot images and molecular-weight information are provided in the accompanying attachment.

### 2.12. Quantitative Real-Time PCR

At 24 h after challenge with 55055 or 46274 (MOI 1; *n* = 4 recorded observations per group, including the uninfected control), RNA was isolated using the RNeasy Mini Kit (QIAGEN, Hilden, Germany, 74104), reverse-transcribed using PrimeScript with gDNA Eraser (Takara, Kusatsu City, Japan, RR047A), and analyzed using PowerUp SYBR Green Master Mix (Thermo Fisher, Vilnius, Lithuania, A25742). Expression was normalized to β-actin and calculated by the 2^−ΔΔCt^ method. Primer sequences are provided in Supplementary Table S1.

### 2.13. Adhesion Assay

To assess adhesion, RAW 264.7 cells were infected with cKP or hvKP (MOI 50 or 100) for 1 h (*n* = 3 recorded observations per strain and MOI), washed three times with sterile PBS, lysed in 0.2% Triton X-100 for 30 min, serially diluted, and plated on LB agar. Adhesion was quantified as recovered CFU/mL. These observations were obtained from within-run wells in one experimental run and were not independent experimental repeats.

### 2.14. Phagocytosis Assay

To assess phagocytosis, RAW 264.7 cells infected with 55055 or 46274 (MOI 50 or 100) for 3 h (*n* = 3 recorded observations per strain and MOI) were washed three times and treated with 50 μg/mL apramycin for 30 min; neither strain grew at this concentration. Cells were washed, lysed in 0.2% Triton X-100 for 30 min, serially diluted, and plated on LB agar. Phagocytosis was quantified as recovered CFU/mL. These observations were also obtained from within-run wells in one experimental run and were not independent experimental repeats.

### 2.15. Single-Cell RNA Sequencing and Bioinformatic Analysis

The pooled-lung single-cell RNA-sequencing libraries were generated from the same terminal-tissue cohort used for H&E, immunohistochemistry, and lung p65 immunofluorescence. PBS lungs were collected on day 4; 55055 lungs on days 1, 2, and 4; and 46274 lungs on days 1 and 2. Three lungs were pooled per condition to generate six composite libraries (PBS; 55055 days 1, 2, and 4; 46274 days 1 and 2). Thus, each condition was represented by one library generated from lungs pooled from three mice (*n* = 3 mice pooled per library; *n* = 1 library per condition), not by independent animal-level libraries. Within 30 min, lungs were kept on ice in sCelLive Tissue Preservation Solution (Singleron), washed, minced, and dissociated in 3 mL sCelLive Tissue Dissociation Solution using the Singleron PythoN system (37 °C, 15 min). Suspensions were filtered (40 μm), treated with GEXSCOPE red blood cell lysis buffer (1:2; 5–8 min, room temperature), centrifuged (300× *g*, 5 min, 4 °C), resuspended in PBS, and assessed by trypan blue. Suspensions (2 × 10^5^ cells/mL) were processed using Singleron Matrix microwell chips and GEXSCOPE Single Cell RNA Library Kits, followed by 150 bp paired-end sequencing on an Illumina NovaSeq 6000 (Illumina, San Diego, CA, USA).

CeleScope v1.9.0 used Cutadapt v1.17 for adapter/poly(A) trimming, STAR v2.6.1a for alignment to mouse GRCm38 (Ensembl 92), and featureCounts v2.0.1 to generate UMI matrices. Cells with <200 genes or gene/UMI counts in the upper 2% were excluded, as were genes detected in <5 cells; 84,009 cells remained. Raw counts were normalized by total counts per cell and log-transformed, and 2000 highly variable genes were selected (seurat_v3). Harmony v0.0.6 integrated the six libraries before Louvain clustering, UMAP visualization, and cluster annotation using cluster-associated genes. Each condition comprised one pooled library from three mice and was not an animal-level replicate.

Differentially expressed genes were identified by scanpy.tl.rank_genes_groups (Scanpy v1.9.3 in Python 3.10; Wilcoxon rank-sum test, default parameters) and required detection in at least 10% of cells in either group, an absolute average log_2_ fold change > 0.25, and *p* < 0.05. GO and KEGG enrichment used clusterProfiler v4.0.0 with Benjamini–Hochberg adjustment (adjusted *p* < 0.05). CellChat v1.6.1 in R and its built-in ligand–receptor database were applied using the recommended workflow to infer putative intercellular signaling networks. Monocle2 v2.10.0 ordered transcriptionally similar cells along pseudotime to describe state relationships, visualized with the plot_cell_trajectory function. UCell v1.1.0 calculated per-cell gene-set scores from ranked expression using the Mann–Whitney U statistic. These single-cell comparisons were descriptive at the library level and were not treated as animal-level inferential tests.

### 2.16. Statistical Analysis

Statistical analyses were performed using GraphPad Prism 10.1.2, except for the single-cell analyses described above. H&E ordinal scores and IHC positive-cell percentages were analyzed separately across the six observed conditions using the Kruskal–Wallis test with tie correction (chi-square approximation, df = 5), followed by Dunn’s test for all 15 pairwise comparisons within each endpoint. Each mouse was the biological unit (*n* = 3 per condition). These exploratory condition-level analyses did not separately estimate strain, time, or strain-by-time effects. Quantitative plots show individual values and mean ± SD unless otherwise stated; H&E medians and ranges are also reported in the text because the scores are ordinal. Survival was compared using the log-rank test; body-weight trajectories were summarized descriptively. Lung-burden values were log10-transformed before between-strain comparisons at the same post-infection day. These comparisons were matched by sampling day, not disease stage. Two-sided Welch’s unequal-variance *t*-test was used for the 55055-versus-46274 comparisons: *p* = 0.000115 on day 1 and *p* = 2.62 × 10^−8^ on day 2. The corresponding sample sizes were *n* = 4 and *n* = 5 for 55055 and 46274 on day 1, respectively, and *n* = 6 per strain on day 2. For the mouse-level p-p65/β-actin ratios (PBS, 55055 day 1, and 46274 day 1; *n* = 3 per group), normality was assessed using the Shapiro–Wilk test. Given the small groups and deviation from normality in the 46274 group, the three independent groups were compared using an exact Kruskal–Wallis test followed by Dunn’s multiple-comparison test, with adjustment across all three pairwise comparisons. For the in vitro assays, the corresponding statistical tests and p values are specified in the legends. For in vitro assays, *n* denotes within-run biological wells or image fields from one experiment unless otherwise stated; these observations are not independent experimental repeats. The in vitro results are interpreted descriptively. Where inferential analysis was appropriate, statistical significance was defined as *p* < 0.05. Pooled-library single-cell comparisons do not provide animal-level inference. H&E scoring, IHC marker quantification, and immunoblot densitometry were performed at the mouse level, whereas p65 nuclear/cytoplasmic localization remains qualitative.

## 3. Results

### 3.1. Study Design

**Figure 1:**
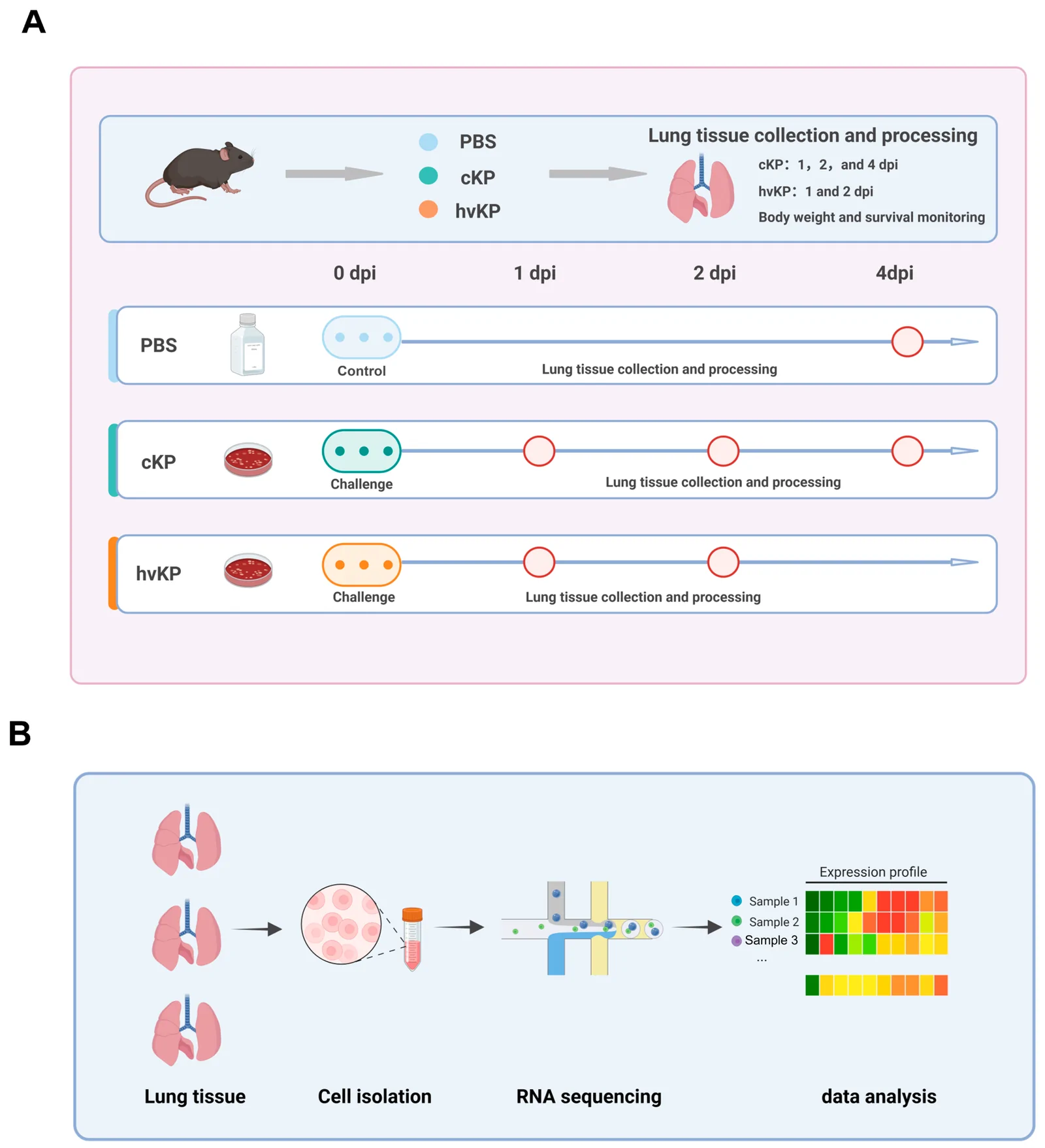
Experimental design and single-cell RNA-sequencing workflow. (**A**) Mice were treated with PBS or challenged with strain 55055 or 46274; the survival/body-weight cohort comprised *n* = 4 mice per infection group, and terminal-tissue cohorts are specified in the Methods and subsequent legends. (**B**) Lung tissues were subjected to single-cell isolation, RNA sequencing, and bioinformatic analysis; each condition comprised one library generated from three pooled mouse lungs (*n* = 3 mice per library; *n* = 1 library per condition). Gray arrows in panel A indicate the experimental sequence, blue arrows indicate the post-infection timeline, and light blue, teal, and orange identify PBS, cKP, and hvKP, respectively; red-outlined circles mark lung-tissue collection time points. Created with BioRender.com.

Female 6–8-week-old C57BL/6 mice received intranasal inoculation of 30 μL containing 6.5 × 10^6^ CFU/mL of strain 55055 or strain 46274 for the final survival/body-weight experiment; this cohort contained *n* = 4 mice per infected group and no contemporaneous PBS group. PBS controls were included in the separate terminal-tissue cohort. Body weight and survival were followed for 7 days; terminal lungs were collected from PBS mice on day 4, from 55055-infected mice on days 1, 2, and 4, and from 46274-infected mice on days 1 and 2 (Figure 1A,B). The same post-infection day was treated as a matched calendar time point, not as evidence that the two groups were at the same disease stage.

### 3.2. Strain 46274 Was Associated with More Severe Disease than Strain 55055 in This Mouse Model

**Figure 2:**
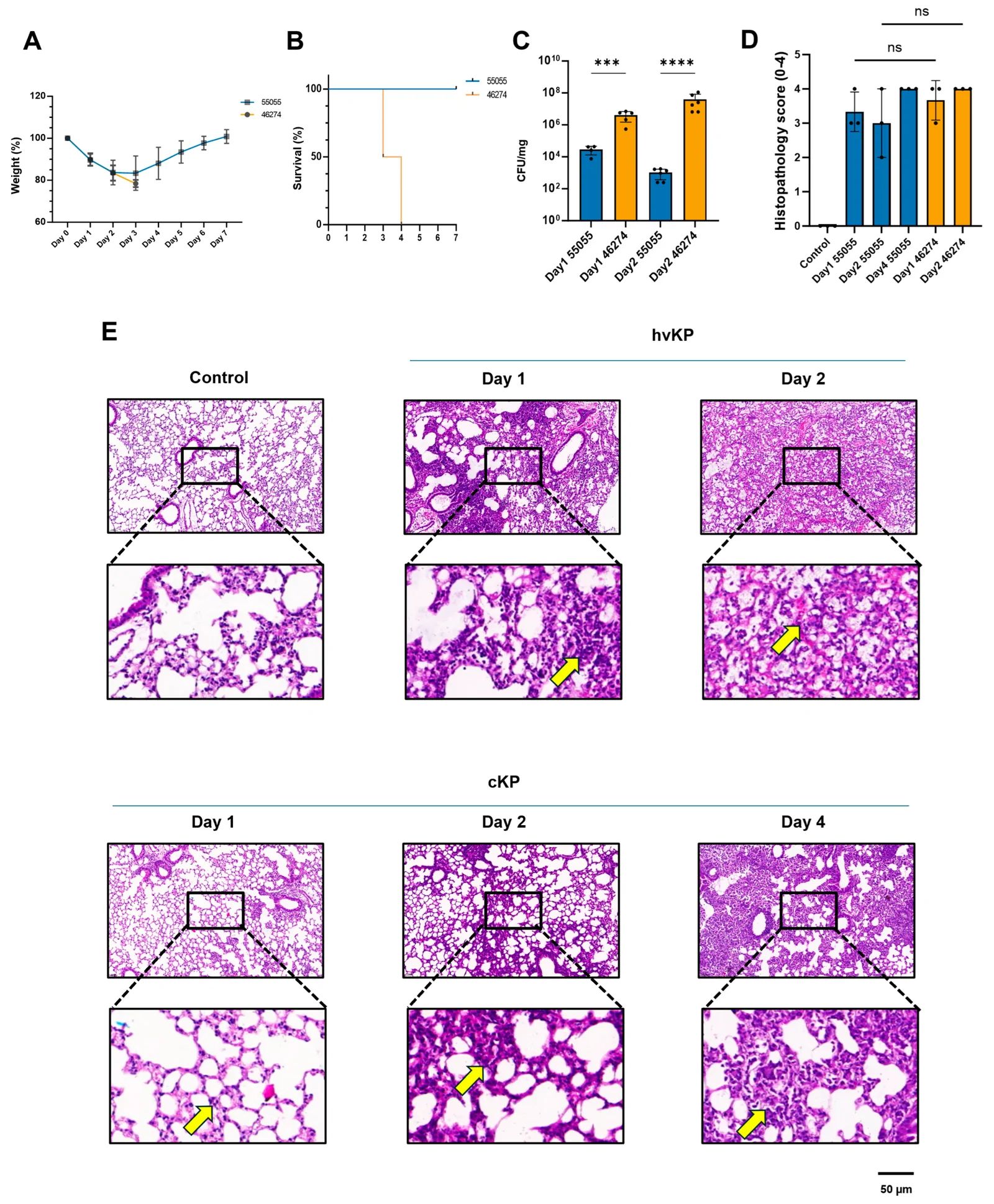
Disease progression in mice after pulmonary infection with strain 55055 or 46274. (**A**) Changes in body weight (mean ± SD; descriptive). (**B**) Kaplan–Meier survival curves (*n* = 4 mice per strain; log-rank *p* = 0.0084). (**C**) Bacterial burdens in lung tissues (mean ± SD): day 1, 55055 *n* = 4 and 46274 *n* = 5; day 2, 55055 *n* = 6 and 46274 *n* = 6. Raw CFU·mg^−1^ values are shown on a logarithmic axis, and log_10_-transformed values were compared using two-sided Welch’s unequal-variance *t*-tests: day 1, *p* = 0.000115 (***); day 2, *p* = 2.62 × 10^−8^ (****). (**D**) H&E injury scores on a 0–4 ordinal scale (0, normal; 1, minimal; 2, mild; 3, moderate; 4, severe). Each dot represents one mouse (*n* = 3 per condition); bars show mean ± SD for descriptive display. Control denotes PBS sampled on day 4; 55055 was sampled on days 1, 2, and 4, and 46274 on days 1 and 2. Tie-corrected Kruskal–Wallis test (chi-square approximation), *H* = 12.1008, df = 5, *p* = 0.0334; Dunn’s test covered all 15 pairwise comparisons, none of which was significant. ns denotes adjusted *p* ≥ 0.05 for the indicated comparisons. (**E**) Representative H&E-stained lung sections and corresponding digital enlargements from the same six conditions. Yellow arrows indicate foci of inflammatory-cell infiltration. Assessor identity and blinding were not documented. For panels (**C**–**E**), strain comparisons at the same post-infection day do not imply matched disease stages. Scale bar, 50 μm.

Mice infected with strain 55055 initially lost weight but recovered, and all animals in the analyzed strain 55055 survival cohort survived. In the strain 46274 group, mean body weight fell below 80% of baseline by day 3; mortality reached 50% on day 3 and 100% by day 4 (*n* = 4/group; Figure 2A,B). Representative H&E sections showed foci of acute and chronic inflammatory-cell infiltration in the lung parenchyma, appearing more extensive after strain 46274 than 55055 infection on day 2 (Figure 2E). This descriptive day-2 comparison was not disease-stage-matched. Scores for 55055 versus 46274 had medians (ranges) of 3 (3–4) versus 4 (3–4) on day 1 and 3 (2–4) versus 4 (4–4) on day 2; all PBS day-4 scores were 0 and all 55055 day-4 scores were 4 (*n* = 3 mice per condition; Figure 2D). The six-condition Kruskal–Wallis test was significant (*H* = 12.1008, df = 5, *p* = 0.0334), but none of the 15 Dunn-adjusted pairwise comparisons was significant (all adjusted *p* ≥ 0.0522). Thus, the quantitative scores did not confirm a specific between-strain difference. The small groups and concentration of scores at the upper limit constrain interpretation, and non-significance does not establish equivalence.

### 3.3. Pulmonary Bacterial Burdens Differed Between Strains and Sampled Time Points

Mean strain 55055 lung burdens were (2.96 ± 1.65) × 10^4^ CFU·mg^−1^ on day 1 (*n* = 4) and (1.03 ± 0.67) × 10^3^ CFU·mg^−1^ on day 2 (*n* = 6)). Corresponding mean burdens for strain 46274 were (4.19 ± 2.72) × 10^6^ CFU·mg^−1^ on day 1 (*n* = 5) and (4.00 ± 4.27) × 10^7^ CFU·mg^−1^ on day 2 (*n* = 6; Figure 2C). The raw CFU·mg^−1^ values are shown on a logarithmic axis, and log_10_-transformed values were compared using a two-sided Welch’s unequal-variance *t*-test: *p* = 0.000115 on day 1 and *p* = 2.62 × 10^−8^ on day 2. Because the study was not burden-matched, subsequent immune-response comparisons cannot separate isolate-associated effects from effects of these markedly unequal pulmonary bacterial loads.

### 3.4. Identification of the Major Cell Populations in Mouse Lung Tissue After Infection

**Figure 3:**
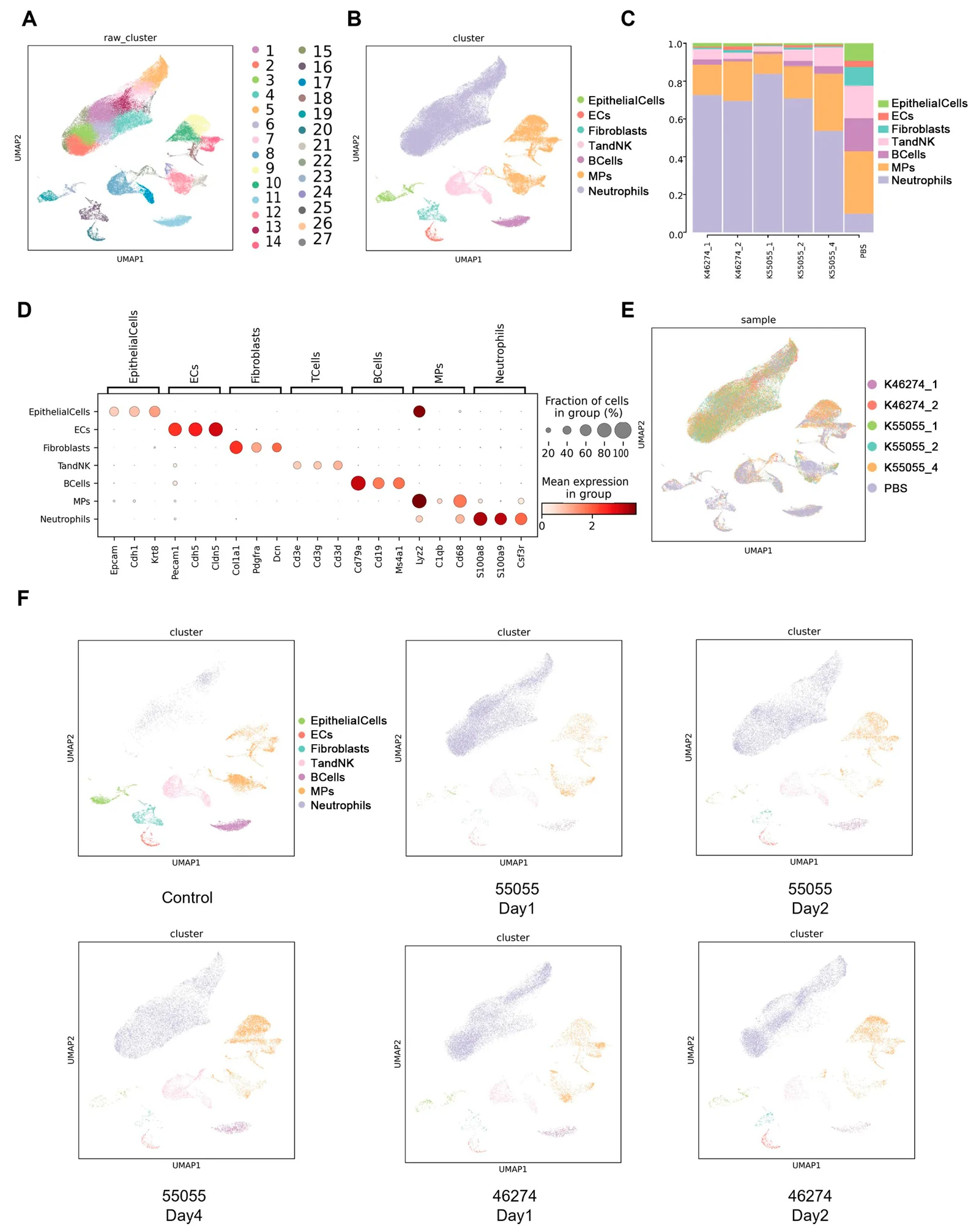
Single-cell analysis of the major cell types in lung tissues. (**A**) UMAP visualization of unsupervised clustering of all cells. (**B**) Annotation of the major cell types. (**C**) Relative proportions of each cell type across experimental groups. (**D**) Expression of representative marker genes in each cell type. (**E**) Integrated UMAP distribution of cells across experimental groups. (**F**) UMAP distributions of the major cell types in each experimental group. Six libraries were analyzed; each condition comprised one library generated from lungs pooled from three mice (*n* = 3 mice pooled per library; *n* = 1 library per condition). Results are descriptive and were not treated as animal-level inferential comparisons.

Six composite libraries (PBS; 55055 on days 1, 2, and 4; 46274 on days 1 and 2; three mice pooled per library) yielded 84,009 filtered cells. Unsupervised clustering identified 27 cell types, which were grouped into seven major populations; marker summaries, a Harmony-integrated UMAP, and condition-stratified UMAPs supported the annotations (Figure 3A,B,D–F). Neutrophils accounted for a larger fraction of cells in infected libraries than in the PBS library (Figure 3C). Each condition was represented by one pooled library (*n* = 3 mice pooled per library; *n* = 1 library per condition); these comparisons are descriptive and do not support animal-level *p* values.

### 3.5. Inferred Intercellular Communication in Pooled Lung Libraries

**Figure 4:**
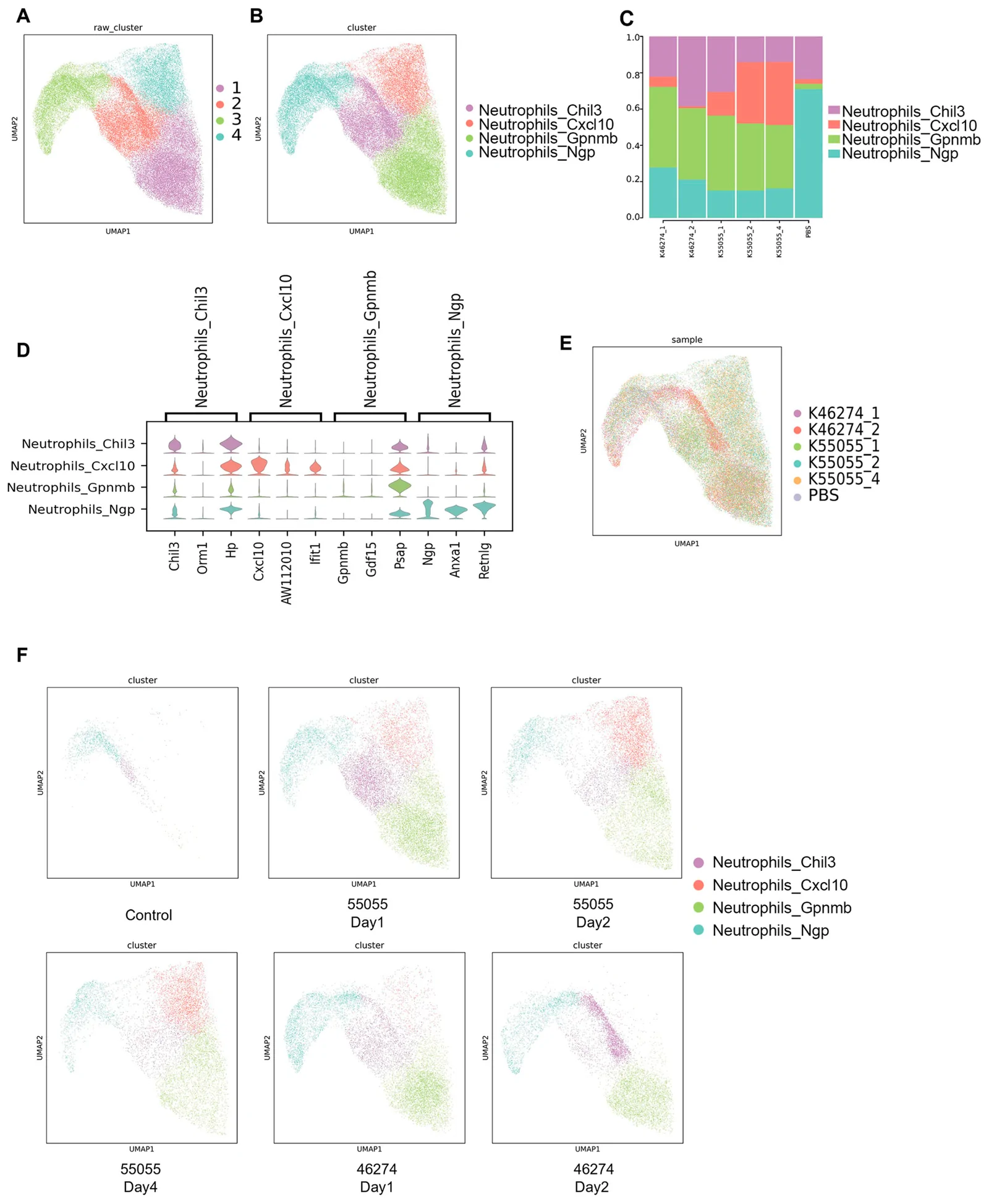
Single-cell analysis of pulmonary neutrophil subsets. (**A**) UMAP visualization of unsupervised neutrophil clustering. (**B**) Annotation of neutrophil subsets. (**C**) Relative proportions of each subset across experimental groups. (**D**) Expression of representative marker genes in each neutrophil subset. (**E**) Integrated UMAP distribution of neutrophils across experimental groups. (**F**) UMAP distributions of neutrophil subsets in each experimental group. Each condition comprised one library generated from lungs pooled from three mice (*n* = 3 mice pooled per library; *n* = 1 library per condition); results are descriptive and not animal-level inferential comparisons. Comparisons between strains on shared sampling days do not imply matched disease stages.

CellChat inferred the most outgoing interactions for fibroblasts. MP-related predictions varied among pooled libraries (Supplementary Figure S1A). Compared with PBS, infected libraries showed lower inferred fibroblast–MP and MP-autocrine strengths but higher neutrophil-autocrine strength (Supplementary Figure S1B). Each condition comprised one pooled library from three mice, precluding assessment of animal-level variability. Pulmonary neutrophil-subset composition is summarized in Figure 4.

### 3.6. Neutrophil-Subset Composition in Pooled Lung Libraries

Unsupervised clustering and marker-expression patterns defined Neutrophils_Chil3, Neutrophils_Cxcl10, Neutrophils_Gpnmb, and Neutrophils_Ngp subsets (Figure 4A,B,D–F). Neutrophils_Ngp formed the largest fraction in the PBS library. Relative to PBS, the infected pooled libraries contained lower Neutrophils_Ngp and higher Neutrophils_Gpnmb fractions. The Neutrophils_Cxcl10 fraction was low in the PBS library and in both strain 46274 libraries, higher in the strain 55055 day-1 library, and highest in the strain 55055 day-2 and day-4 libraries. At the same calendar days 1 and 2, which were not assumed to represent matched disease stages, the strain 55055 pooled libraries contained larger Neutrophils_Cxcl10 fractions than the corresponding strain 46274 libraries (Figure 4C). These patterns describe the sampled libraries, not replicated changes over time.

### 3.7. Mononuclear Phagocyte Subset Composition in Pooled Lung Libraries

**Figure 5:**
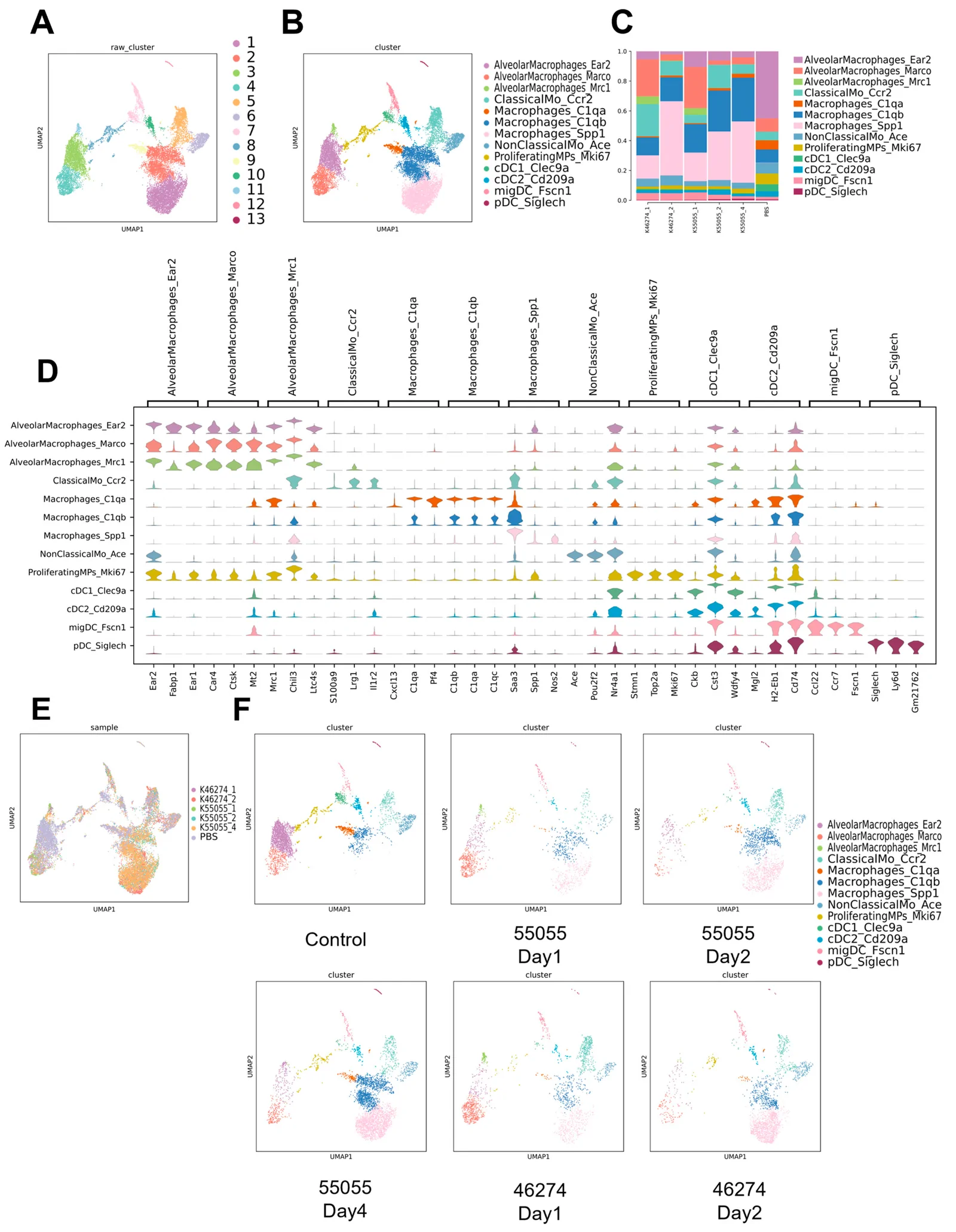
Single-cell analysis of pulmonary mononuclear phagocyte subsets. (**A**) UMAP visualization of unsupervised mononuclear phagocyte clustering. (**B**) Annotation of mononuclear phagocyte subsets. (**C**) Relative proportions of each subset across experimental groups. (**D**) Expression of representative marker genes in each mononuclear phagocyte subset. (**E**) Integrated UMAP distribution of mononuclear phagocytes across experimental groups. (**F**) UMAP distributions of mononuclear phagocyte subsets in each experimental group. Each condition comprised one library generated from lungs pooled from three mice (*n* = 3 mice pooled per library; *n* = 1 library per condition); results are descriptive and not animal-level inferential comparisons. The day-2 SPP1^+^ macrophage comparison reflects the same sampling day, not matched disease stages.

Unsupervised clustering and marker profiles defined 13 MP subsets (Figure 5A,B,D–F): AlveolarMacrophages_Ear2, AlveolarMacrophages_Marco, AlveolarMacrophages_Mrc1, ClassicalMo_Ccr2, Macrophages_C1qa, Macrophages_C1qb, Macrophages_Spp1, NonClassicalMo_Ace, ProliferatingMPs_Mki67, cDC1_Clec9a, cDC2_Cd209a, migDC_Fscn1, and pDC_Siglech. AlveolarMacrophages_Ear2 formed the largest fraction in the PBS library. The day-1 infected pooled libraries contained relatively larger Marco/Mrc1 fractions; the libraries collected at later post-infection time points contained lower C1qa and higher C1qb/Spp1 fractions. On post-infection day 2, Macrophages_Spp1 occupied a larger fraction of the 46274 than the 55055 pooled library (Figure 5C); this same-day comparison was not disease-stage-matched. These comparisons reflect individual pooled libraries, not replicated temporal changes.

### 3.8. Inferred Communication Among Macrophage Subpopulations

CellChat analysis revealed broadly connected but library-dependent communication among pulmonary myeloid subsets (Supplementary Figure S3A,B). In the Control library, interactions involving AlveolarMacrophages_Ear2 and Neutrophils_Ngp were relatively prominent. Across infected libraries, neutrophil–macrophage connections remained evident, whereas the involvement of C1qa-, C1qb-, Spp1-, and alveolar-macrophage states varied among the sampled libraries. These descriptive networks do not distinguish strain-associated differences from differences in disease progression. With one pooled library from three mice per condition, these inferred networks require independent validation.

### 3.9. Tissue-Level CD206, CD86, and Ly6G Staining After Infection

**Figure 6:**
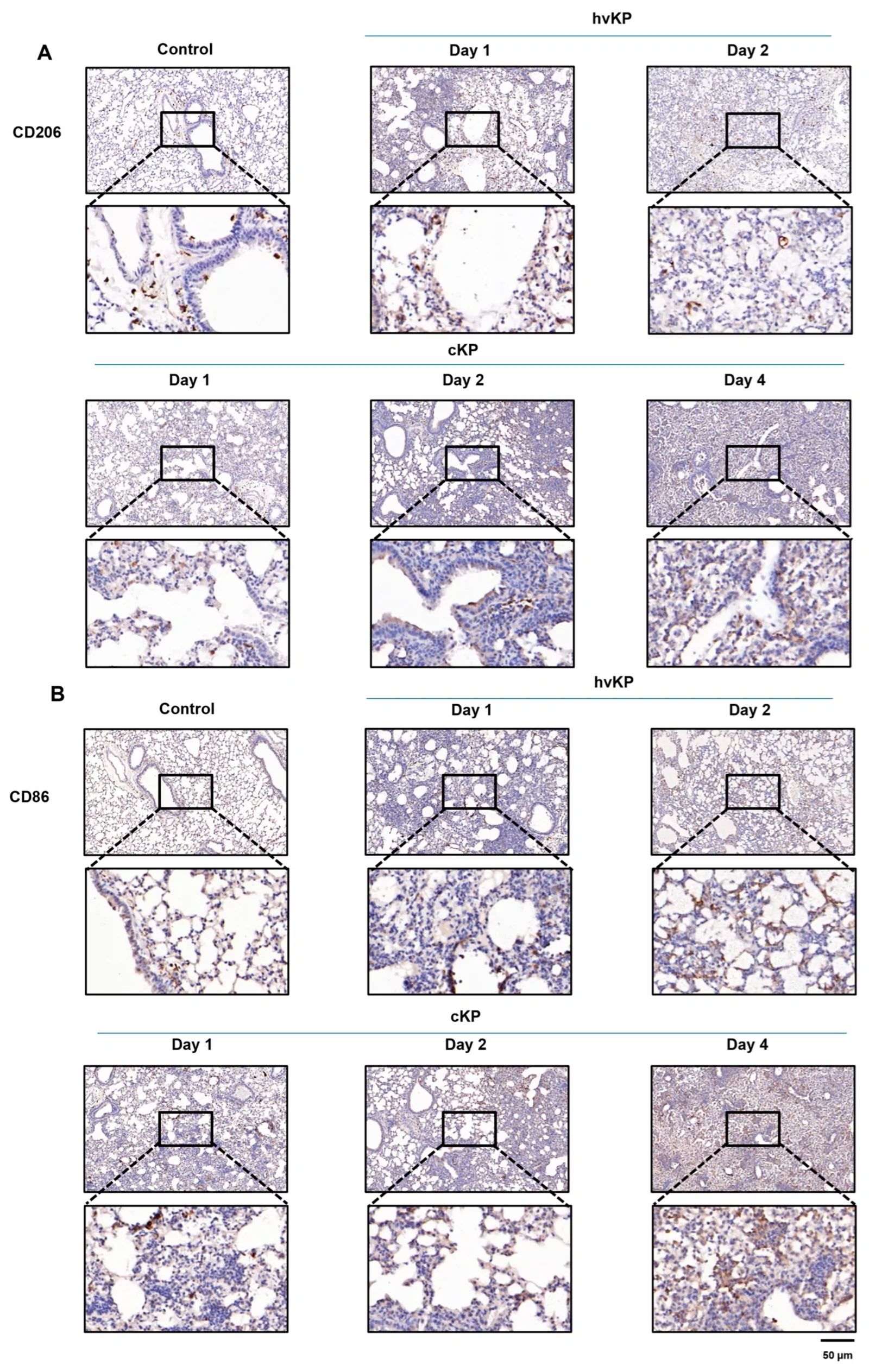

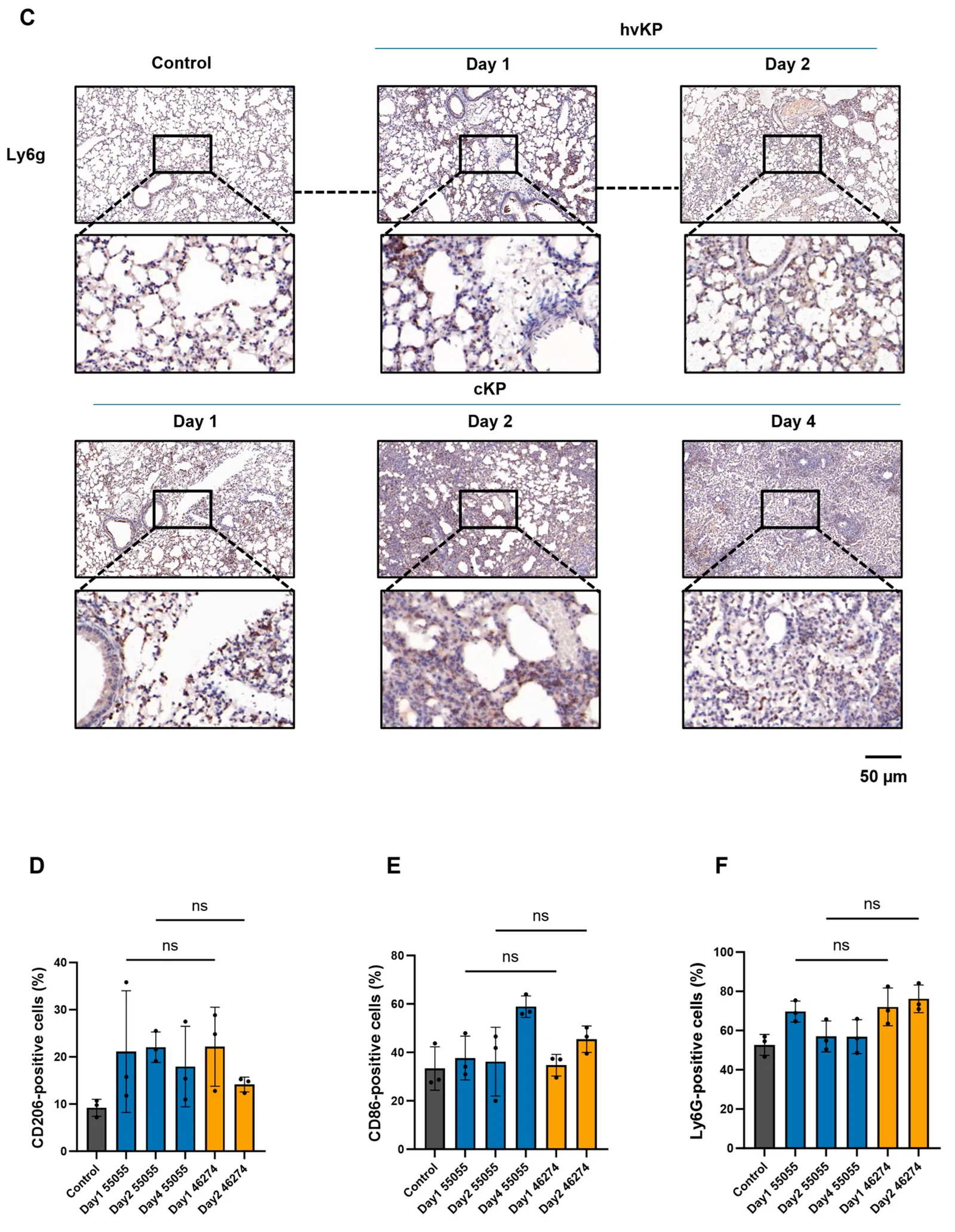
Lung immunohistochemistry and mouse-level quantification following infection with strain 55055 or 46274. (**A**–**C**) Representative CD206, CD86, and Ly6G staining, respectively, with corresponding digital enlargements. (**D**–**F**) Positive-cell percentages for CD206, CD86, and Ly6G, respectively, calculated as positive cells among all cells. Each dot represents one mouse (*n* = 3 per condition); bars show mean ± SD. Control denotes PBS sampled on day 4; 55055 was sampled on days 1, 2, and 4, and 46274 on days 1 and 2. Separate tie-corrected Kruskal–Wallis tests across the six conditions (chi-square approximation, df = 5) gave *p* = 0.0992 for CD206, *p* = 0.0798 for CD86, and *p* = 0.0434 for Ly6G. Dunn’s test covered all 15 pairwise comparisons within each marker; none was significant. ns denotes adjusted *p* ≥ 0.05 for the indicated comparisons. These tissue-level measurements do not identify macrophage subsets; no macrophage-marker co-localization was performed. Blinding and field selection were not documented. Same-day strain comparisons are not disease-stage-matched comparisons. Scale bars, 50 μm.

Representative day-2 sections suggested more CD86-positive and fewer CD206-positive cells after strain 46274 than 55055 infection (Figure 6A–C). The day-2 comparison was based on sampling time and was not matched by disease stage. Mouse-level positive-cell percentages are shown for all six conditions (*n* = 3 mice per condition; Figure 6D–F). The overall Kruskal–Wallis tests were not significant for CD86 (*H* = 9.8421, df = 5, *p* = 0.0798) or CD206 (*H* = 9.2573, df = 5, *p* = 0.0992), whereas the Ly6G test was significant (*H* = 11.4327, df = 5, *p* = 0.0434). No pairwise comparison for any marker remained significant after Dunn adjustment across all 15 comparisons within each marker. On day 2, median (range) values for 55055 versus 46274 were 41.85% (19.97–46.59%) versus 46.51% (39.47–50.24%) for CD86, 21.57% (19.10–25.50%) versus 14.82% (12.36–15.25%) for CD206, and 54.90% (50.43–65.67%) versus 73.39% (70.98–84.14%) for Ly6G. These numerical patterns do not establish strain-specific differences or equivalence. The measurements describe tissue-level marker abundance, not M1/M2 macrophage proportions, because no macrophage-marker co-localization was performed.

### 3.10. Whole-Lung NF-κB-Associated Findings After Infection

**Figure 7:**
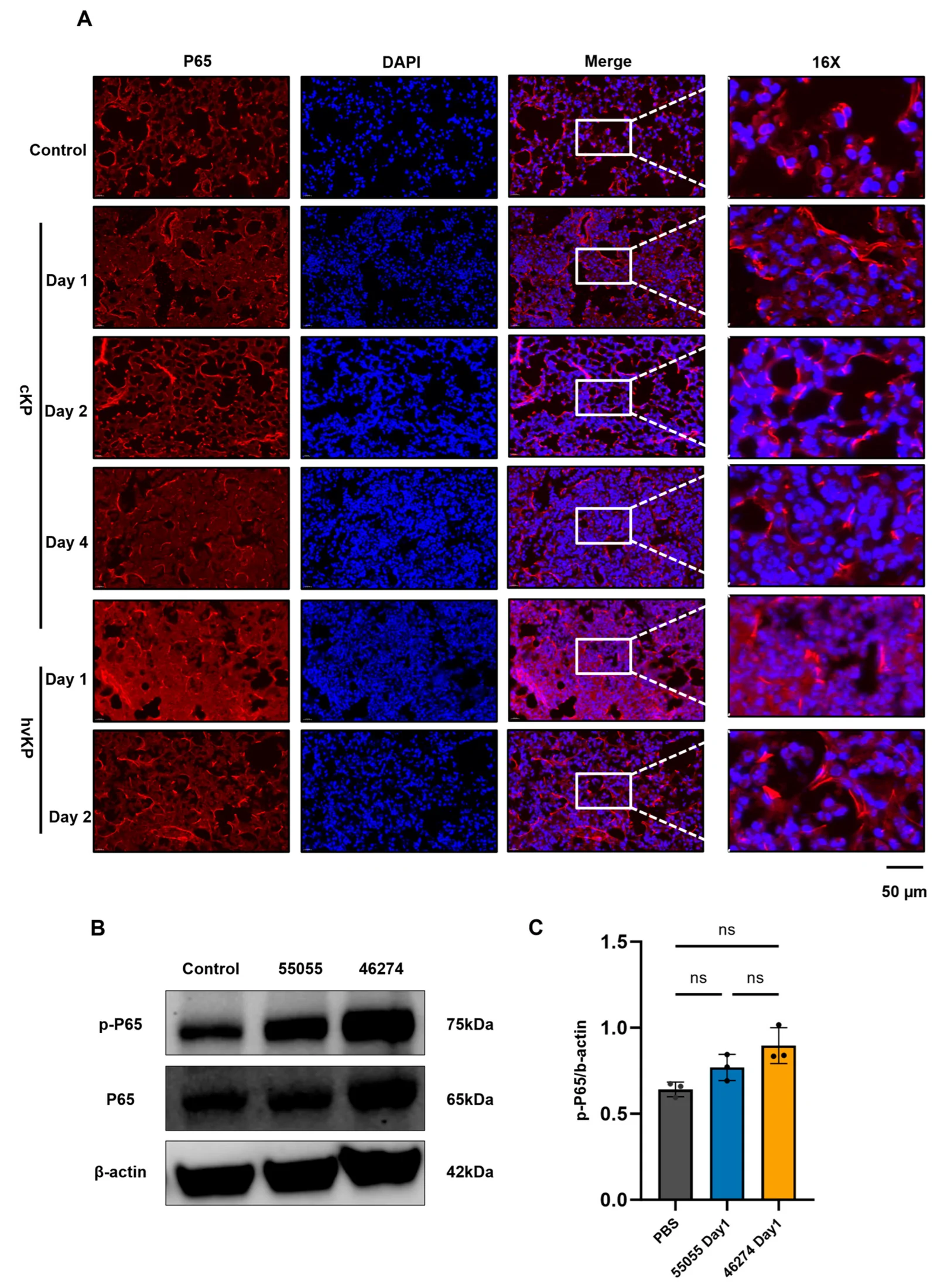
Lung p65 immunofluorescence and Western-blot analysis following infection with strain 55055 or 46274. (**A**) Representative p65 immunofluorescence from three mice per group; nuclear/cytoplasmic localization was not quantified. Scale bars, 50 μm. (**B**) Representative whole-lung immunoblots for phospho-p65, total p65 and β-actin; infected samples were collected on day 1. (**C**) Mouse-level phospho-p65/β-actin ratios. Each dot represents one independent mouse (*n* = 3 per group); bars and error bars indicate mean ± SD. Control denotes PBS. Exact Kruskal–Wallis test, *H* = 5.956, *p* = 0.0250; Dunn-adjusted pairwise *p* = 0.3032 (PBS versus 55055), 0.0512 (PBS versus 46274), and 1.0000 (55055 versus 46274). None of the pairwise comparisons was significant. Original uncropped blots and molecular-weight information are provided in the accompanying attachment. ns, not significant.

Representative day-1 lung immunoblots for phospho-p65, total p65, and β-actin are shown in Figure 7B, and mouse-level p-p65/β-actin ratios are shown in Figure 7C (PBS, 55055, and 46274; *n* = 3 per group). The ratios were numerically higher in the 46274 group. The exact Kruskal–Wallis test gave *H* = 5.956 and *p* = 0.0250. Dunn-adjusted *p* values for all three pairwise comparisons were 0.3032 (PBS versus 55055), 0.0512 (PBS versus 46274), and 1.0000 (55055 versus 46274); none of the pairwise comparisons reached statistical significance. These data therefore do not establish a strain-specific difference in p-p65/β-actin. Figure 7A suggested greater p65 nuclear localization after strain 46274; nuclear/cytoplasmic localization was not quantified. Taken together, these findings are NF-κB-associated rather than evidence that pathway activation caused the pulmonary phenotype; causal inference would require quantitative localization and pathway perturbation.

### 3.11. Lower Cell-Associated Bacterial Readouts with Strain 46274 in RAW 264.7 Cells

**Figure 8:**
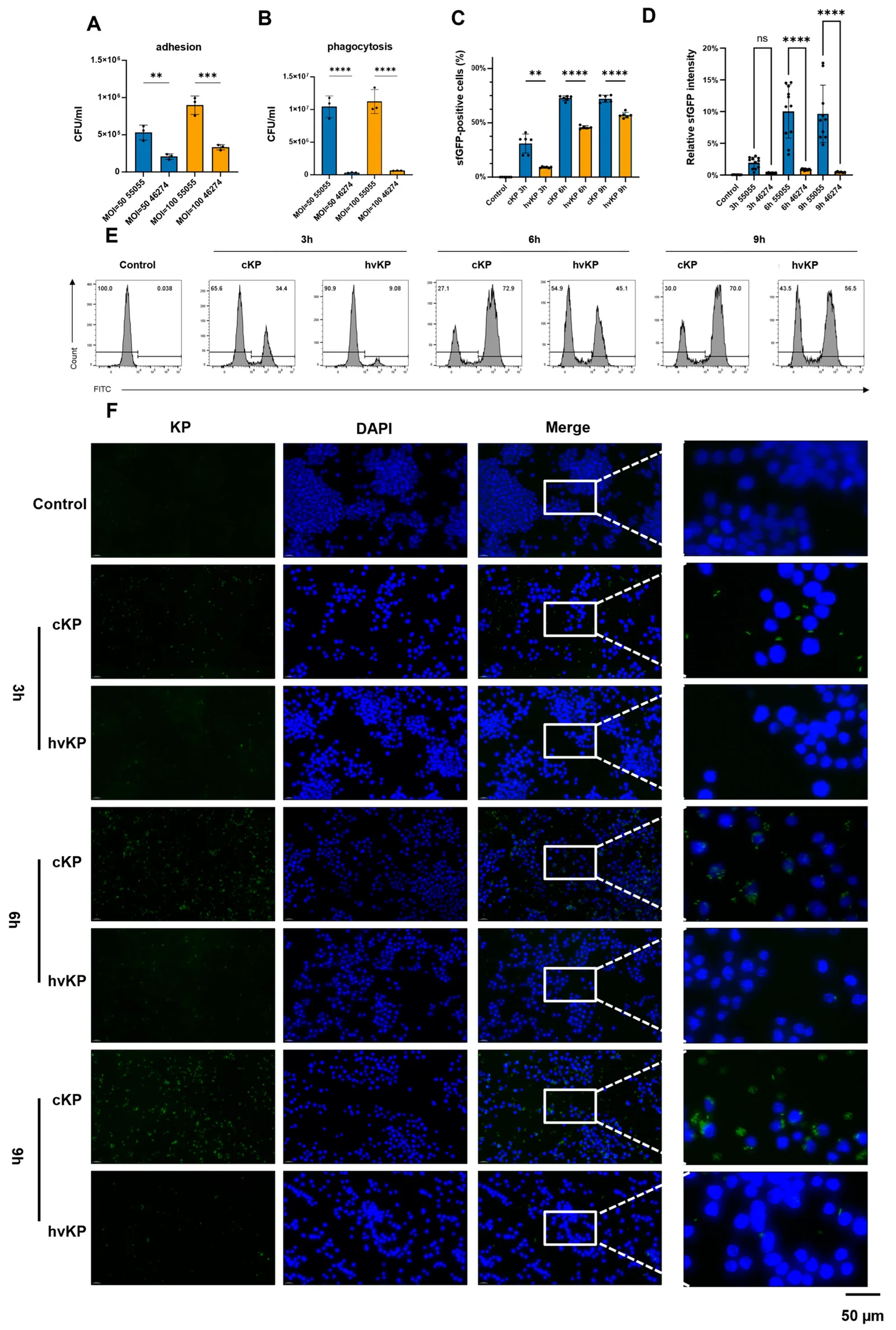
Adhesion- and phagocytosis-associated bacterial readouts in RAW 264.7 cells exposed to strain 55055 or 46274. (**A**) Cell-associated CFU after the adhesion assay (*n* = 3 within-run wells per strain/condition from one experiment); ordinary one-way ANOVA, *F*(3,8) = 38.88, *p* = 4.07 × 10^−5^; Tukey-adjusted strain-comparison *p* = 0.0064 and 0.0002 at the two inoculum conditions shown. (**B**) Apramycin-protected CFU (*n* = 3 within-run wells per strain/condition from one experiment); panel (**B**) is presented descriptively. (**C**) sfGFP-positive cells by flow cytometry: control *n* = 6; 55055/46274 *n* = 6/6, 6/5, and 6/6 at 3, 6, and 9 h. Brown–Forsythe and Welch ANOVA with Dunnett T3 multiple comparisons yielded adjusted *p* = 0.0045, <0.0001, and <0.0001. (**D**) Relative sfGFP intensity: control *n* = 6; 55055/46274 *n* = 11/6, 11/12, and 10/6 at 3, 6, and 9 h. An observation-level one-way ANOVA reported *F*(6,55) = 28.15, *p* < 0.0001; Tukey-adjusted *p* = 0.8946, <0.0001, and <0.0001. Panels (**A**–**D**) show within-run biological wells or image fields from one experiment; the observation-level *p* values do not establish reproducibility across independent experiments. (**E**) Representative flow-cytometry histograms. (**F**) Representative fluorescence images; *Klebsiella pneumoniae* is shown in green, and nuclei are shown in blue. Scale bar, 50 μm. Control denotes uninfected cells. Data are mean ± SD. For panels (A) and (C), symbols denote: ** *p* < 0.01; *** *p* < 0.001; **** *p* < 0.0001. Panels (**B**) and (**D**) are shown without inferential significance annotations and are interpreted descriptively. Because the sfGFP-labeled derivatives were not directly benchmarked against their parental isolates, panels (**C**–**F**) do not establish functional equivalence between labeled and parental strains. ns, not significant.

At 1 h, recovered cell-associated CFU were lower for strain 46274 than for strain 55055 (Figure 8A). The wash-and-recovery endpoint does not distinguish surface-bound from internalized bacteria. Apramycin-protected CFU were also numerically lower for 46274 (Figure 8B). Flow cytometry and microscopy showed lower cell-associated sfGFP readouts for 46274 at the indicated time points (Figure 8C–F). These fluorescence endpoints do not by themselves establish bacterial internalization. The displayed adhesion, phagocytosis, flow-cytometry, and microscopy data were generated in one experimental run; their wells and image fields should not be equated with independent experimental repeats. Because the sfGFP-labeled derivatives were not benchmarked against their parental isolates by growth-curve or inoculum CFU-equivalence testing, these fluorescence results apply to the labeled derivatives and do not establish functional equivalence to the parental strains.

### 3.12. Higher IL-6 and TNF-α Readouts After Strain 46274 Exposure

**Figure 9:**
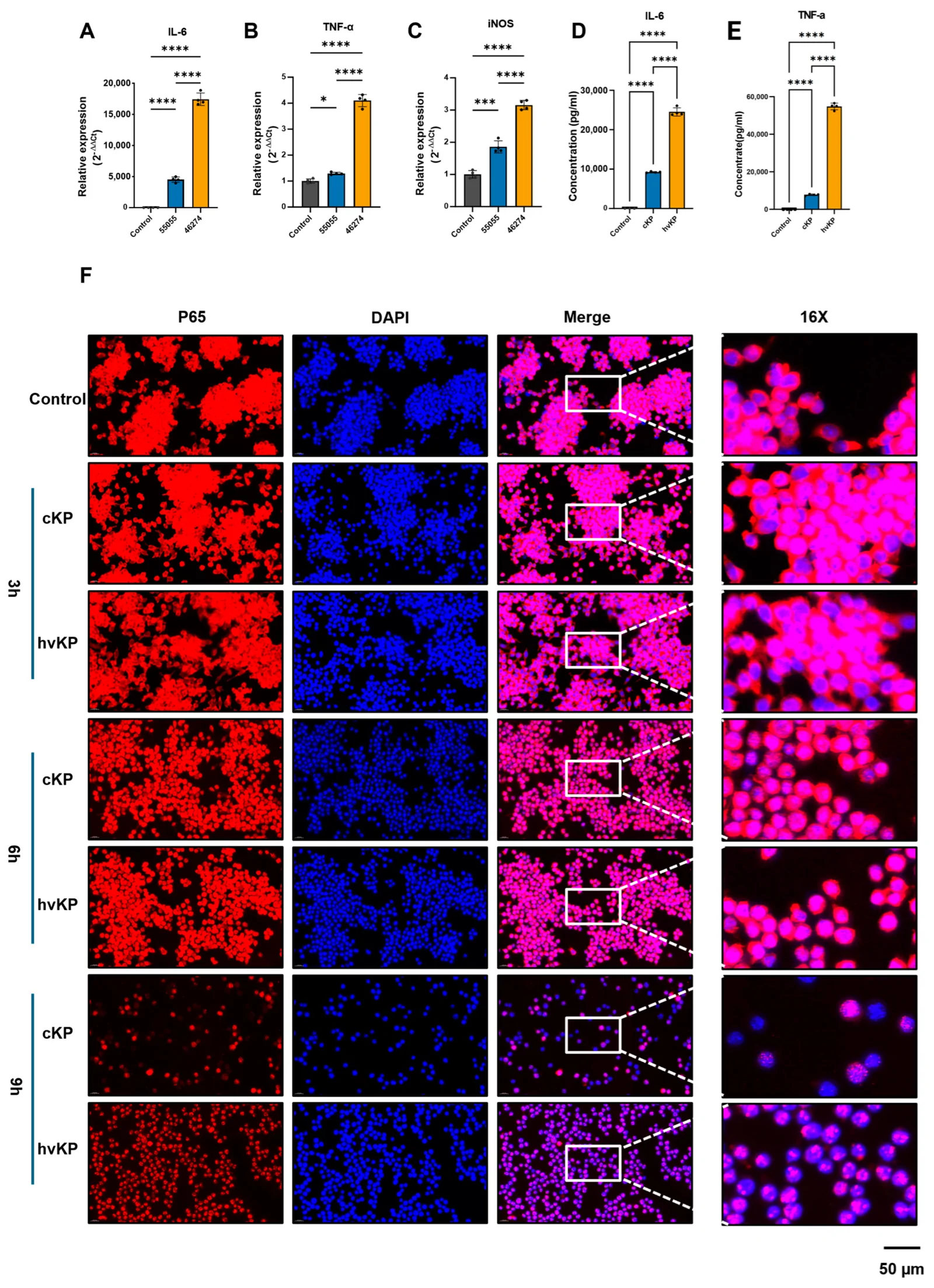
Inflammatory responses and qualitative p65 localization in RAW 264.7 cells exposed to strain 55055 or 46274. (**A**) Relative IL-6 mRNA at 24 h (MOI 1; *n* = 4/condition); ordinary one-way ANOVA, *F*(2,9) = 835.8, *p* < 0.0001, with all Tukey-adjusted pairwise *p* < 0.0001. (**B**) Relative TNF-α mRNA at 24 h (MOI 1; *n* = 4/condition); ordinary one-way ANOVA, *F*(2,9) = 553.9, *p* < 0.0001, with Tukey-adjusted *p* = 0.0481 for control versus 55055 and *p* < 0.0001 for the other pairwise comparisons. (**C**) Relative iNOS mRNA at 24 h (MOI 1; *n* = 4/condition); ordinary one-way ANOVA, *F*(2,9) = 228.9, *p* < 0.0001, with Tukey-adjusted *p* = 0.0004 for control versus 55055 and *p* < 0.0001 for the other pairwise comparisons. (**D**) Supernatant IL-6 at 24 h (MOI 1; control *n* = 3, 55055 *n* = 4, 46274 *n* = 4); ordinary one-way ANOVA, *F*(2,8) = 1307, *p* < 0.0001, with all Tukey-adjusted pairwise *p* < 0.0001. (**E**) Supernatant TNF-α at 24 h (MOI 1; *n* = 4/condition); ordinary one-way ANOVA, *F*(2,9) = 3163, *p* < 0.0001, with all Tukey-adjusted pairwise *p* < 0.0001. (**F**) Representative immunofluorescence images of intracellular p65 (red) and DAPI (blue) at 3, 6, and 9 h after infection (MOI 50). The available 3 h and 6 h observations did not show a clear group-separating pattern, whereas the 9 h images suggested greater p65 nuclear localization after strain 46274 exposure. The panels were assembled using a common display/contrast setting. Control denotes cells processed in parallel without bacteria. p65 nuclear/cytoplasmic localization was not quantified. Scale bar, 50 μm. Data are presented as mean ± SD; A–C were analyzed directly as 2^−ΔΔCt^ values. Symbols indicate the reported thresholds: * *p* < 0.05; *** *p* < 0.001; **** *p* < 0.0001. For panels (**A**–**E**), *n* denotes within-run biological wells from one experiment; these values do not represent independent experimental repeats.

At 24 h, RAW 264.7 cells exposed to strain 46274 had higher relative IL-6, TNF-α, and iNOS mRNA levels and higher supernatant IL-6 and TNF-α concentrations (pg/mL) than cells exposed to strain 55055 (Figure 9A–E). The displayed qPCR and ELISA data were generated in one experimental run using within-run biological wells and do not represent independent experimental repeats. For the qPCR panels and the TNF-α ELISA, *n* = 4 recorded wells were analyzed per group; for the IL-6 ELISA, the uninfected control had *n* = 3 and each bacterial-strain group had *n* = 4. Accordingly, these findings are preliminary, are limited to the RAW 264.7 model, and do not establish reproducibility across independent experiments.

### 3.13. Qualitative p65 Localization in Infected RAW 264.7 Macrophages

Representative images at 3, 6, and 9 h are shown in Figure 9F. The available 3 h and 6 h observations did not show a clear group-separating pattern, whereas the 9 h images suggested more pronounced p65 nuclear localization after strain 46274 than strain 55055 exposure. The panels were assembled using a common display/contrast setting. No quantitative nuclear/cytoplasmic analysis was performed, so this imaging result remains a qualitative NF-κB-associated pattern.

### 3.14. Descriptive Features of SPP1^+^ Macrophages in Pooled Lung Libraries

**Figure 10:**
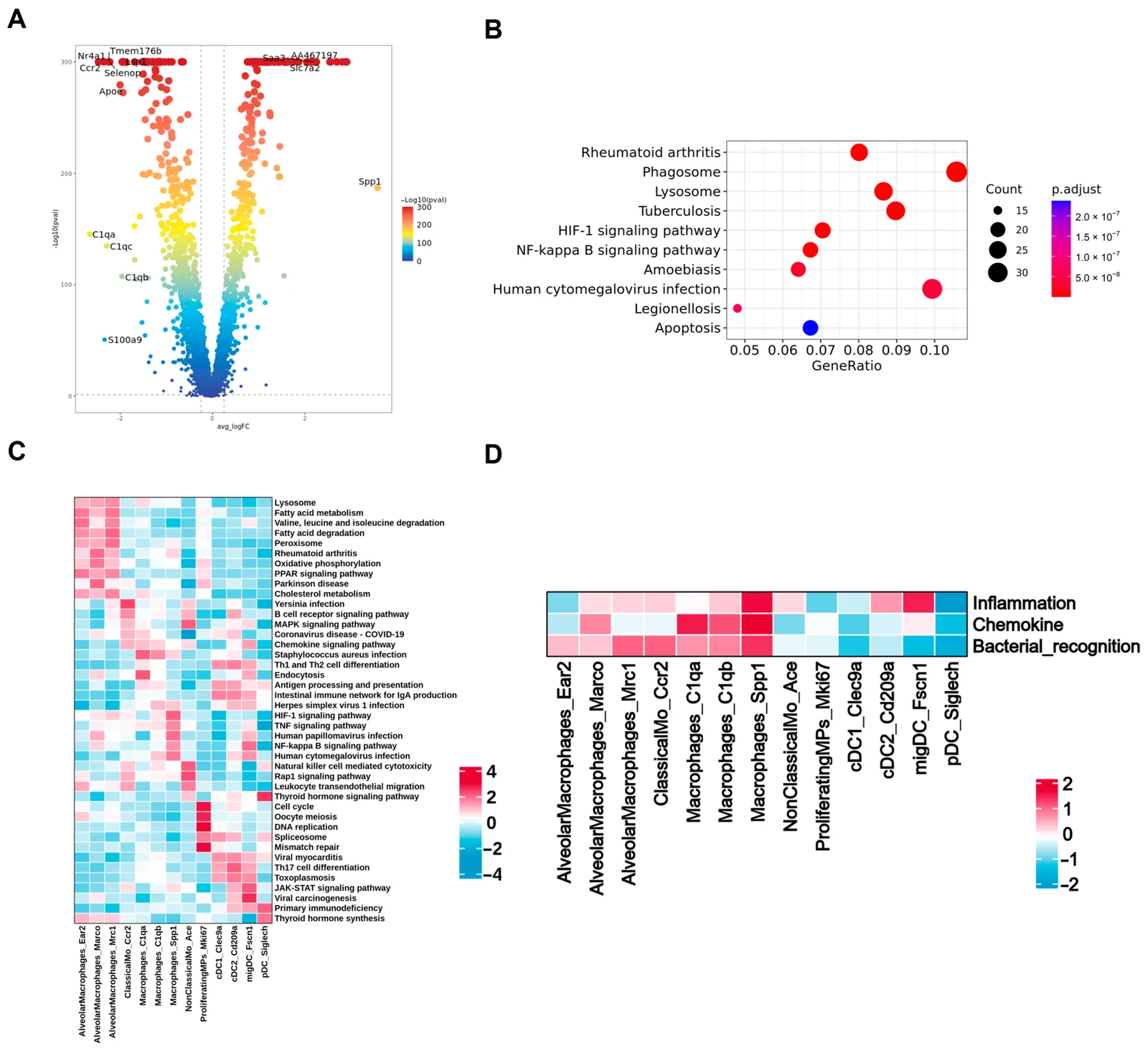
Transcriptional and functional characteristics of Macrophages_Spp1. (**A**) Volcano plot of differentially expressed genes between Macrophages_Spp1 and other mononuclear phagocyte (MP) subsets. (**B**) KEGG enrichment analysis of genes highly expressed in Macrophages_Spp1. (**C**) KEGG pathway enrichment scores across MP subsets. (**D**) Inflammation, chemokine, and bacterial-recognition signature scores across MP subsets. Red and blue indicate relatively high and low scores, respectively. Each condition was represented by one library generated from lungs pooled from three mice (*n* = 3 mice pooled per library; *n* = 1 library per condition); results are descriptive and not animal-level inferential comparisons.

On post-infection day 2, SPP1^+^ macrophages formed a larger fraction of the 46274 than the 55055 pooled library (Figure 5C). These libraries were collected at the same post-infection time point but were not matched by disease stage. Differentially expressed genes in SPP1^+^ macrophages are shown in Figure 10A; enriched terms included phagosome, lysosome, HIF-1, NF-κB, and apoptosis pathways (Figure 10B). KEGG-associated score profiles varied across macrophage subsets (Figure 10C). CellChat-inferred interaction counts varied among pooled libraries (Supplementary Figure S2). With one pooled library from three mice per condition, these findings require validation in independent animals. They do not establish an animal-level reproducible difference or a mechanistic role for SPP1^+^ macrophages.

### 3.15. Functional Gene-Set Scoring Across Pulmonary Mononuclear Phagocyte Subsets

Among monocyte-derived macrophage subsets, Macrophages_C1qa and Macrophages_C1qb showed high chemokine and bacterial-recognition scores, whereas Macrophages_Spp1 showed high scores across all three modules and high TNF- and NF-κB-associated gene-set scores (Supplementary Figure S4A,B; Figure 10D), consistent with an inflammation- and pathogen-recognition-associated transcriptional state. With one pooled library from three mice per condition, these scores cannot establish reproducible animal-level differences.

Among alveolar macrophage subsets, AlveolarMacrophages_Ear2 showed low inflammation-response and chemokine module scores, AlveolarMacrophages_Marco showed a moderate chemokine score, and AlveolarMacrophages_Mrc1 showed a high bacterial-recognition score (Figure 10D).

### 3.16. Pseudotime Analysis of Pulmonary Macrophage States

Computational pseudotime placed Ccr2 cells at earlier positions, C1qb cells at intermediate positions, and C1qa/Spp1-associated cells at terminal positions on separate branches. Ace and Adgre5 expression was lower at later positions. In the 46274 pooled-library ordering, Bin2 expression was lower, Apoe expression was higher at later positions, and AA467197, Arg1, and Basp1 expression was higher at intermediate-to-late positions (Supplementary Figure S5A–D).

For alveolar macrophages, the 55055 pooled-library pseudotime ordering displayed a bifurcating trajectory, in which a shared segment split into two arms. In contrast, the 46274 pooled-library ordering was unbranched. Within this ordering, Abcg1 expression progressively decreased, whereas Acp5 expression increased. AA467197 and Acod1 showed marked increases at later inferred positions. These computational orderings do not establish lineage relationships or directionality (Supplementary Figure S6A–D).

### 3.17. Pathway Enrichment Analysis of Alveolar Macrophages

GO enrichment analysis for AlveolarMacrophages_Marco identified terms related to antigen processing and presentation, cytokine-mediated signaling, responses to interferon-γ and interferon-β, leukocyte migration and chemotaxis, phagocytic vesicles, proteasome and peptidase complexes, and MHC class I protein binding (Supplementary Figure S7A).

GO enrichment analysis for AlveolarMacrophages_Ear2 identified terms related to regulation of cell and cell–cell adhesion, actin filament organization, leukocyte migration, myeloid and mononuclear cell differentiation, the cell cortex, focal adhesions, membrane microdomains, actin binding, and GTPase regulation (Supplementary Figure S7B).

GO enrichment analysis for AlveolarMacrophages_Mrc1 identified terms related to lipid catabolism, receptor-mediated endocytosis, and negative regulation of phosphate metabolism, phosphorylation, immune-system processes, and leukocyte/cell activation; peroxisomal, lysosomal, lytic-vacuole, and clathrin-coated compartments; and phospholipid, phosphatidylinositol, and carbohydrate binding (Supplementary Figure S7C). KEGG pathway-score heatmaps for the Marco^+^, Ear2^+^, and Mrc1^+^ subsets across the six pooled libraries are shown in Supplementary Figure S7D–F, respectively.

## 4. Discussion

The in vivo and in vitro findings were directionally concordant with respect to inflammatory-associated readouts. At the same inoculum, strain 46274 was associated with higher pulmonary bacterial burdens and greater mortality than strain 55055. Representative inflammatory-cell infiltration appeared more extensive. Although the six-condition H&E and Ly6G omnibus tests were significant, no specific pairwise comparison remained significant after Dunn adjustment; the CD86 and CD206 omnibus tests were not significant. Whole-lung p-p65/β-actin ratios were numerically higher in the 46274 group, but the strain comparison was not significant after Dunn adjustment (adjusted *p* = 1.0000). p65 nuclear/cytoplasmic localization was not quantified. These results support cautious, associative interpretation of the inflammatory-associated pattern rather than a claim of macrophage polarization or pathway activation. Likewise, RAW 264.7 cells exposed to strain 46274 showed higher IL-6, TNF-α, and iNOS readouts and more pronounced late p65 nuclear localization. At the same time, strain 46274 showed lower adhesion and phagocytosis in RAW 264.7 cells. These observations are not contradictory because lung CFU reflects net bacterial growth and clearance in an intact host, whereas the in vitro assays measure adhesion and phagocytosis at defined time points. Inflammatory readouts therefore need not vary in parallel with these processes. Because the study was not burden-matched, the pulmonary immune differences cannot be separated from effects of the markedly unequal bacterial burdens, and they should not be attributed solely to intrinsic properties of the two isolates. The p65 localization images remain qualitative, and the overall NF-κB-associated findings do not establish a causal pathway.

The lower adhesion and phagocytosis observed for strain 46274 are compatible with studies showing that capsule production and hypermucoviscosity can reduce phagocyte uptake in particular hvKP backgrounds [8,32,33]. Other isolate-specific findings illustrate how immune-evasion phenotypes depend on bacterial background and experimental context. In NTUH-K2044, OmpK35/36 may modulate virulence through direct or indirect effects on capsular-polysaccharide export; in ATCC 43816, OxyR supports oxidative-stress resistance and systemic virulence [34,35]. The cited liver-abscess review additionally notes that K1/K2 capsule types show high resistance to Kupffer-cell clearance [36]. Conversely, Depo16-mediated capsule removal increased susceptibility to peritoneal macrophages, reduced bacterial burdens, and prolonged survival in a mouse bacteremia model [37], whereas benzoic acid reduced capsule production and hypermucoviscosity, impaired resistance to phagocytosis and other host defenses, and improved survival in infected larvae [38]. A previous cKP–hvKP macrophage comparison reported reduced hvKP uptake together with lower cytokine responses and cell toxicity [26]. The present strain pair showed a similar direction for adhesion and phagocytosis but a different direction for inflammatory-mediator production. Differences in isolate genotype, inoculum, sampling time, and assay endpoint could contribute to this distinction; because capsule abundance and the cited bacterial factors were not measured or perturbed here, they should be regarded as possible explanations rather than demonstrated mechanisms.

The stronger inflammatory-associated readouts observed with strain 46274 broadly agree with a report linking hvKP exposure to NF-κB-related signaling [23]. In an intestinal model, itaconate-mediated SYK inhibition suppressed hvKP-associated inflammation [39], whereas hvKP-derived outer-membrane vesicles were reported to induce inflammatory responses [40]. In a bloodstream/liver-injury model, nicotinamide mononucleotide reduced inflammatory injury [41]. Pulmonary transcriptomic work also identified TNF- and IL-17-related response signatures [42]. STAT1-dependent PANoptosis of alveolar macrophages was associated with hvKP-induced lung injury in another strain and model [22]. Together, these studies show that macrophage-associated inflammatory responses and tissue injury vary with infection site, bacterial strain, disease stage, and intervention, but they do not, as a group, establish a host-protective effect of macrophage inflammation. The present data are compatible with inflammatory involvement but differ from mechanistic studies. p65 nuclear/cytoplasmic localization was not quantified, and the numerical increase in whole-lung p-p65/β-actin was not supported by a significant strain comparison after multiple-comparison adjustment. This ratio does not quantify p65 nuclear localization or establish a change in the fraction of p65 that is phosphorylated. Neither NF-κB nor cell death was experimentally perturbed, and the tissue measurements were not macrophage-specific.

The clinical relevance of these findings should be interpreted cautiously. Although the isolates were recovered from bronchoalveolar lavage fluid, their clinical origin does not establish that the host responses measured here occur in patients. WHO surveillance documented the emergence of carbapenemase-producing hvKP [6], providing clinical context rather than validation of the present inflammatory mechanism. This study examined one isolate pair in female C57BL/6 mice at a defined inoculum. Thus, the higher burden, mortality, and NF-κB-associated readouts observed with strain 46274 represent a strain- and model-specific phenotype rather than a universal feature of human hvKP pneumonia. Validation in additional patient isolates, both sexes, burden-matched models, primary human or airway macrophages, and clinical samples will be required.

The larger fraction of SPP1^+^ macrophages in the day-2 strain 46274 pooled library provides a further hypothesis-generating observation from a same-day comparison without disease-stage matching. In murine *K. pneumoniae* pneumonia, global osteopontin deficiency impaired early neutrophil recruitment and increased later bacterial burdens and mortality, suggesting a host-protective role for osteopontin without demonstrating a macrophage-specific effect [29]. In experimental SARS-CoV-2 pneumonia, Slamf9^+^ macrophages expressing Spp1 were associated with viral clearance and subsequent resolution of inflammation [30]. By comparison, the SPP1^+^ population observed here occurred in the higher-burden, more severe bacterial-infection library and had relatively high inflammation-, chemokine-, and bacterial-recognition-module scores. This difference in disease context prevents assignment of either a protective or pathogenic role. Because each condition was represented by one pooled library and SPP1^+^ macrophages were not selectively depleted or manipulated, the result identifies a candidate cell state for validation rather than a mediator of lung injury.

This study integrates an equal-inoculum pneumonia model and descriptive pooled-lung single-cell profiling in the same clinical-isolate pair. Complementary macrophage assays assessed adhesion, phagocytosis, and inflammatory readouts. These approaches distinguish pulmonary disease severity, adhesion and phagocytosis measurements, and inflammatory-associated readouts, which need not vary together. The study describes SPP1^+^ macrophages as a hypothesis-generating candidate component of the strain-associated pulmonary response that requires validation in independent animals. It does not establish that reduced adhesion or phagocytosis causes greater in vivo persistence, that SPP1^+^ macrophages drive pathology, or that NF-κB is the central mechanism.

The detailed limitations of the present study are summarized in Section 5.

## 5. Study Limitations

Several limitations narrow these conclusions. This exploratory study included only one strain pair and female mice; strain 55055 cannot represent cKP as a whole, and strain 46274 cannot represent all hvKP backgrounds. The design was not burden-matched. The final survival/body-weight experiment lacked a contemporaneous PBS control and used small groups. Sampling times were asymmetric because mortality prevented day-4 sampling of the 46274 group. Consequently, matched calendar days do not necessarily represent matched disease stages. In particular, the day-2 immune findings may reflect different stages of disease progression, and statistical analysis cannot remove this biological confounding. Each single-cell condition comprised one library pooled from three mice, precluding estimation of animal-level variability; CellChat and pseudotime analyses are exploratory and do not demonstrate signaling activity, lineage relationships, or causality. Animal-level H&E scoring, tissue-level CD206/CD86/Ly6G quantification, and day-1 Western-blot densitometry were reported. H&E and Ly6G showed significant omnibus differences across the six observed conditions, but no Dunn-adjusted pairwise comparison was significant; CD86 and CD206 omnibus tests were not significant. These exploratory analyses do not separate strain, time, and interaction effects, and the PBS day-4 group is not a time-matched control for infected day-1/day-2 groups. For p-p65/β-actin, the exact overall Kruskal–Wallis test was significant, but none of the Dunn-adjusted pairwise comparisons was significant. Only three mice per group were available for WB analysis, limiting normality assessment and statistical power; the non-significant pairwise results do not establish equivalence. We plan to repeat the WB analysis in a larger independent mouse cohort to assess the reproducibility of the observed numerical pattern. In this study, the identity of the H&E assessor, blinding status, and method of field selection were not documented; the small H&E groups and concentration of scores at the upper limit constrain detection of severity differences and do not establish equivalence. Blinding of IHC quantification was also not established. No p65 nuclear/cytoplasmic quantification, macrophage co-localization, sfGFP-parental growth/CFU-equivalence validation, or primary alveolar-macrophage/BMDM validation is presented. RAW 264.7 cells therefore do not fully represent primary pulmonary macrophages. Future studies should include additional independently selected isolates, both sexes, animal-level single-cell replication, burden-matched comparisons, larger prospectively powered pathology and immunostaining cohorts, validated fluorescent derivatives, primary lung macrophages, and targeted perturbation of the proposed cell states and pathways. The strain design also confounds sequence type, capsule, resistance determinants, and other background differences. The CFU adhesion/phagocytosis assays were not normalized to cell number or protein. Unequal lung-burden variances and the lack of independent in vitro experimental repeats further limit statistical interpretation.

Overall, this strain- and model-specific comparison describes NF-κB-associated inflammatory readouts and a hypothesis-generating SPP1^+^ macrophage signal in the pooled strain 46274 lung library. The unequal lung burdens and unmatched disease stages prevent attribution of the observed immune differences solely to intrinsic properties of the two isolates, and these observations do not establish mechanisms that distinguish cKP from hvKP as broader clinical categories. Independent animal-level replication, burden-matched comparisons, primary-macrophage validation, and pathway- or cell-state-perturbation studies are required.

## Data Availability

The data supporting the findings of this study are available from the corresponding author upon reasonable request.

## Supplementary Materials

The following supporting information can be downloaded at https://www.mdpi.com/article/10.3390/microorganisms14102337/s1. Figure S1: CellChat-inferred intercellular communication among seven major lung cell populations (EpithelialCells, ECs, Fibroblasts, TandNK, BCells, MPs, and Neutrophils) across six pooled libraries. (A) Interaction-number heatmaps and (B) interaction-weight/strength networks for PBS (Control), 55055 day 1, day 2, and day 4, and 46274 day 1 and day 2; Figure S2: CellChat-inferred interaction-number matrices among major lung cell populations after resolving the pulmonary mononuclear-phagocyte compartment into the displayed subsets across six pooled libraries; Figure S3: CellChat-inferred communication among 13 pulmonary mononuclear phagocyte subsets and four neutrophil subsets across six pooled libraries. (A) Interaction-number matrices and (B) interaction-strength networks for PBS (Control), 55055 days 1, 2, and 4, and 46274 days 1 and 2. Each condition was represented by one library pooled from three mice; analyses are descriptive; Figure S4: TNF and NF-κB signaling pathway scores across 13 pulmonary mononuclear phagocyte subsets. (A) TNF signaling pathway scores. (B) NF-κB signaling pathway scores; Figure S5: Computational pseudotime analysis of five pulmonary monocyte–macrophage states: ClassicalMo_Ccr2, Macrophages_C1qa, Macrophages_C1qb, Macrophages_Spp1, and NonClassicalMo_Ace. (A) Cell-state and pseudotime trajectories for the 55055-infected group. (B) Selected gene-expression trends along pseudotime for the 55055-infected group. (C) Cell-state and pseudotime trajectories for the 46274-infected group. (D) Selected gene-expression trends along pseudotime for the 46274-infected group; Figure S6: Computational pseudotime analysis of three pulmonary alveolar-macrophage states: AlveolarMacrophages_Ear2, AlveolarMacrophages_Marco, and AlveolarMacrophages_Mrc1. (A) Cell-state and pseudotime trajectories for the 55055-infected group. (B) Selected gene-expression trends along pseudotime for the 55055-infected group. (C) Cell-state and pseudotime trajectories for the 46274-infected group. (D) Selected gene-expression trends along pseudotime for the 46274-infected group; Figure S7: Functional enrichment analysis of Marco^+^, Ear2^+^, and Mrc1^+^ alveolar-macrophage subsets. (A–C) Gene Ontology enrichment dot plots for the Marco^+^, Ear2^+^, and Mrc1^+^ subsets, respectively, showing biological process, cellular component, and molecular function categories. (D–F) Heatmaps of KEGG pathway scores for the corresponding subsets across pooled libraries; Table S1: Primer sequences for qRT-PCR analysis.
