# Supplementary material for: Pulmonary Disease and NF-κB-Associated Responses in Mice Infected with Two Klebsiella pneumoniae Isolates
Source: Microorganisms. 2026 Oct 13;14(10):2337. doi: 10.3390/microorganisms14102337 (PMC13707559; doi:10.3390/microorganisms14102337)
Supplement: Supplementary file 1 [file microorganisms-14-02337-s001.zip › microorganisms-4574864-supplementary.pdf]

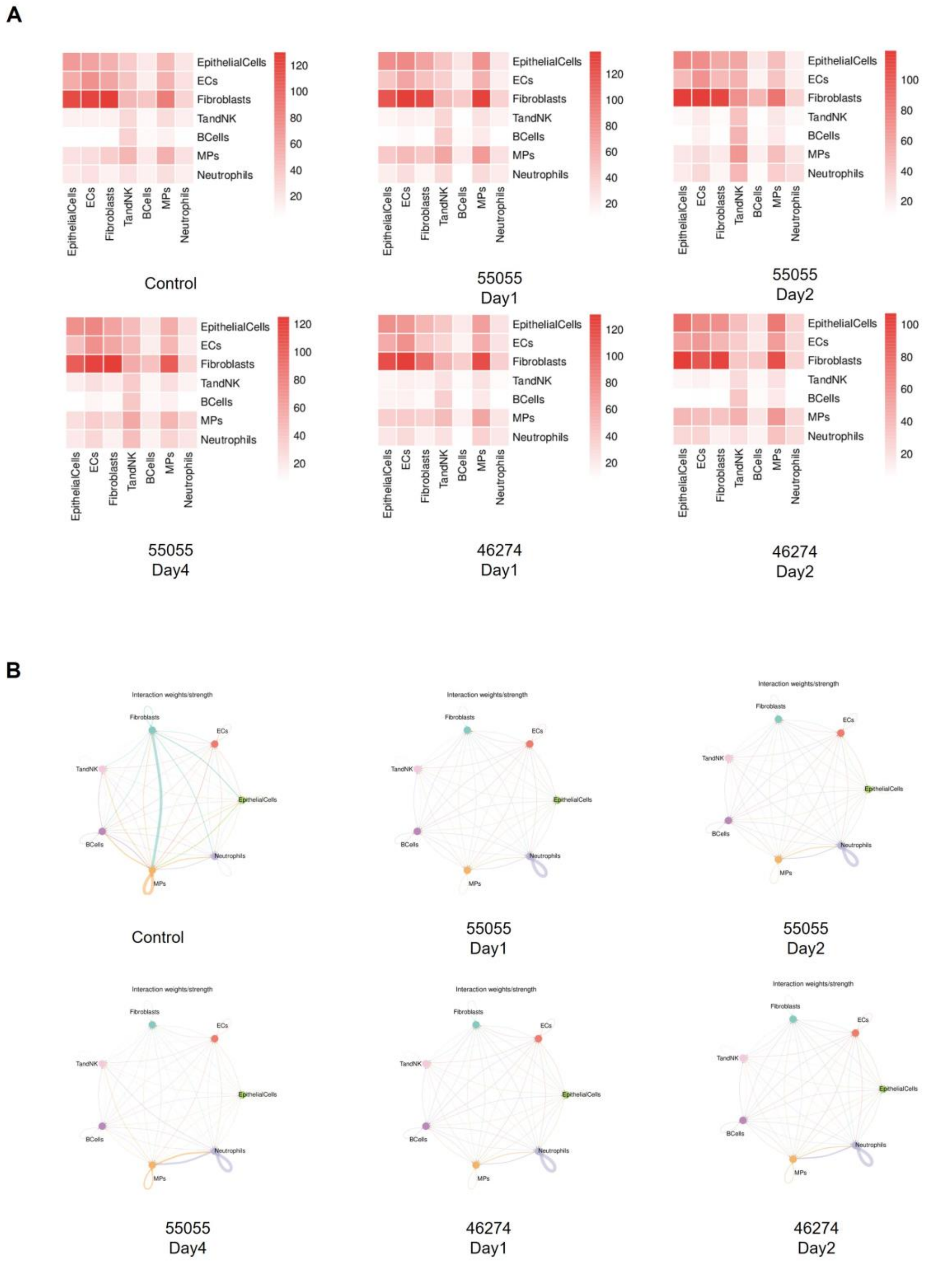


**Supplemental Figure S1.** CellChat-inferred intercellular communication among seven major lung cell populations (EpithelialCells, ECs, Fibroblasts, TandNK, BCells, MPs, and Neutrophils) across six pooled libraries. (A) Interaction-number heatmaps and (B) interaction-weight/strength networks for PBS (Control), 55055 day 1, day 2, and day 4, and 46274 day 1 and day 2.

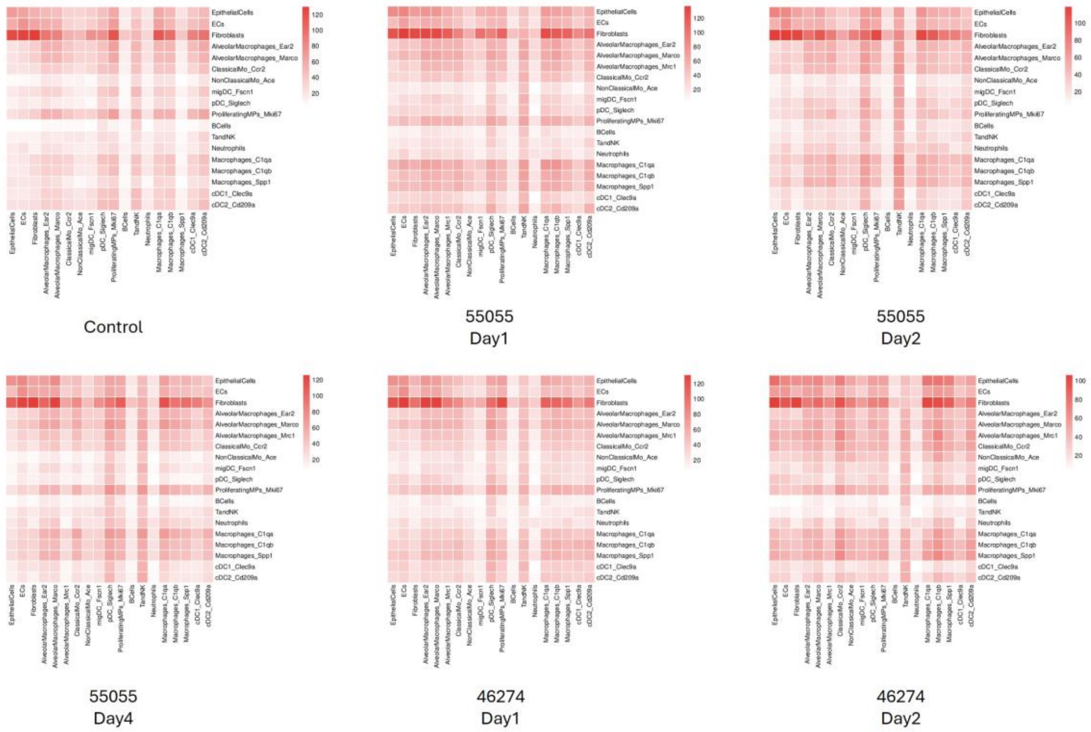


**Supplemental Figure S2.** CellChat-inferred interaction-number matrices among major lung cell populations after resolving the pulmonary mononuclear-phagocyte compartment into the displayed subsets across six pooled libraries.

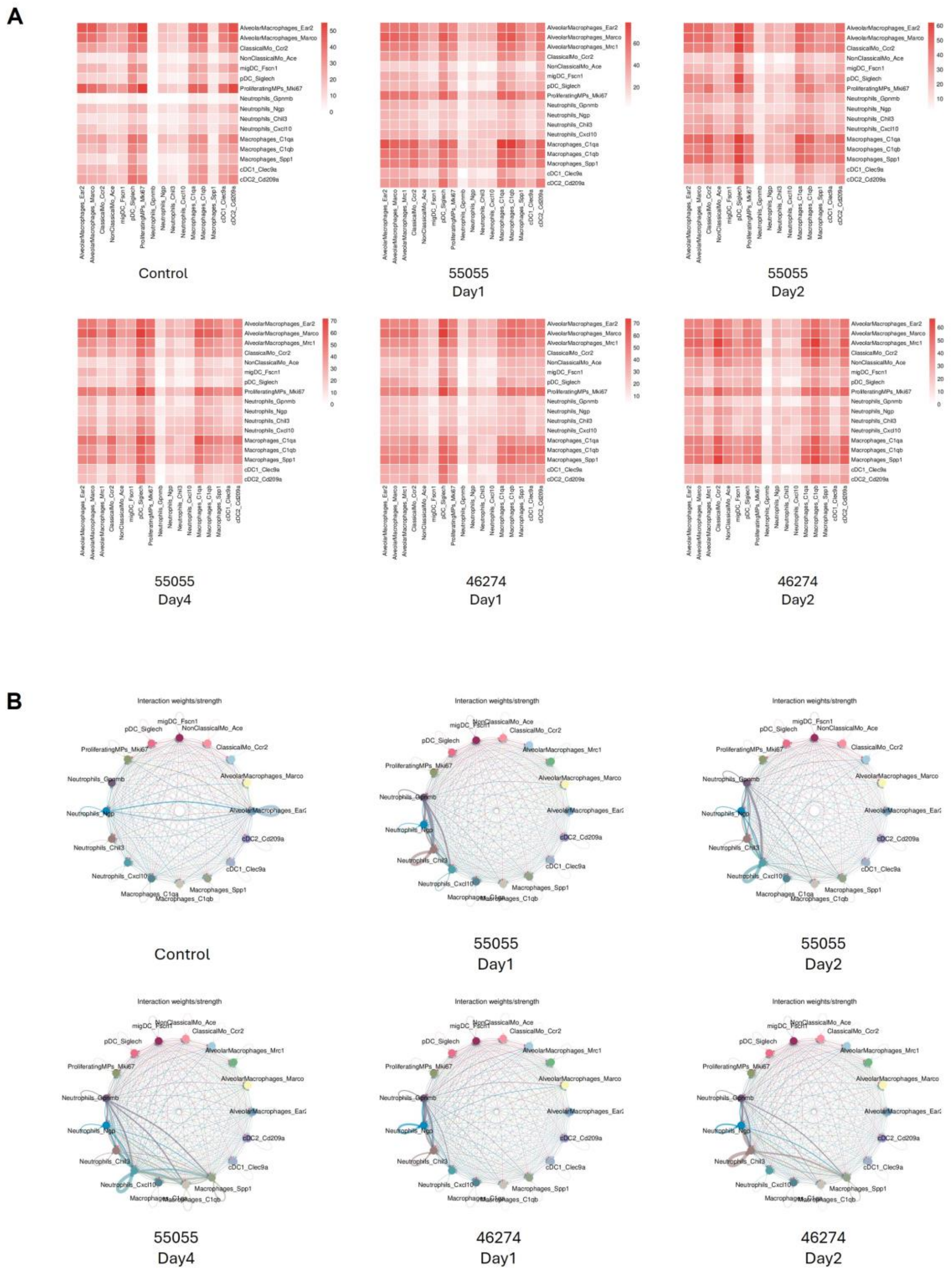


**Supplemental Figure S3.** CellChat-inferred communication among 13 pulmonary mononuclear phagocyte subsets and four neutrophil subsets across six pooled libraries. (A) Interaction-number matrices and (B) interaction-strength networks for PBS (Control), 55055 days 1, 2, and 4, and 46274 days 1 and 2. Each condition was represented by one library pooled from three mice; analyses are descriptive.

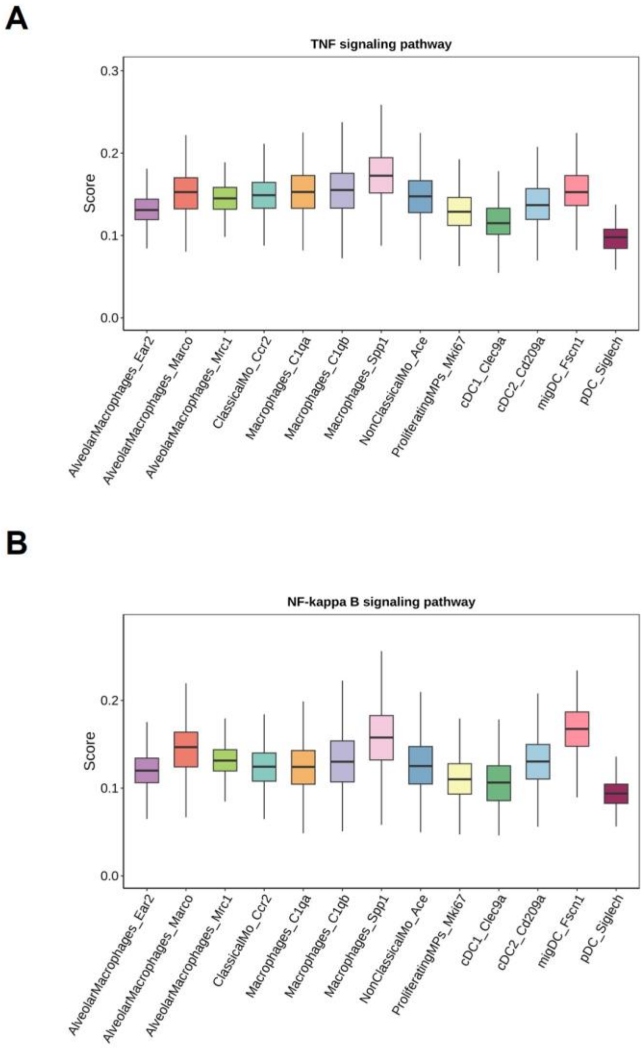


**Supplemental Figure S4.** TNF and NF-κB signaling pathway scores across 13 pulmonary mononuclear phagocyte subsets. (A) TNF signaling pathway scores. (B) NF-κB signaling pathway scores.

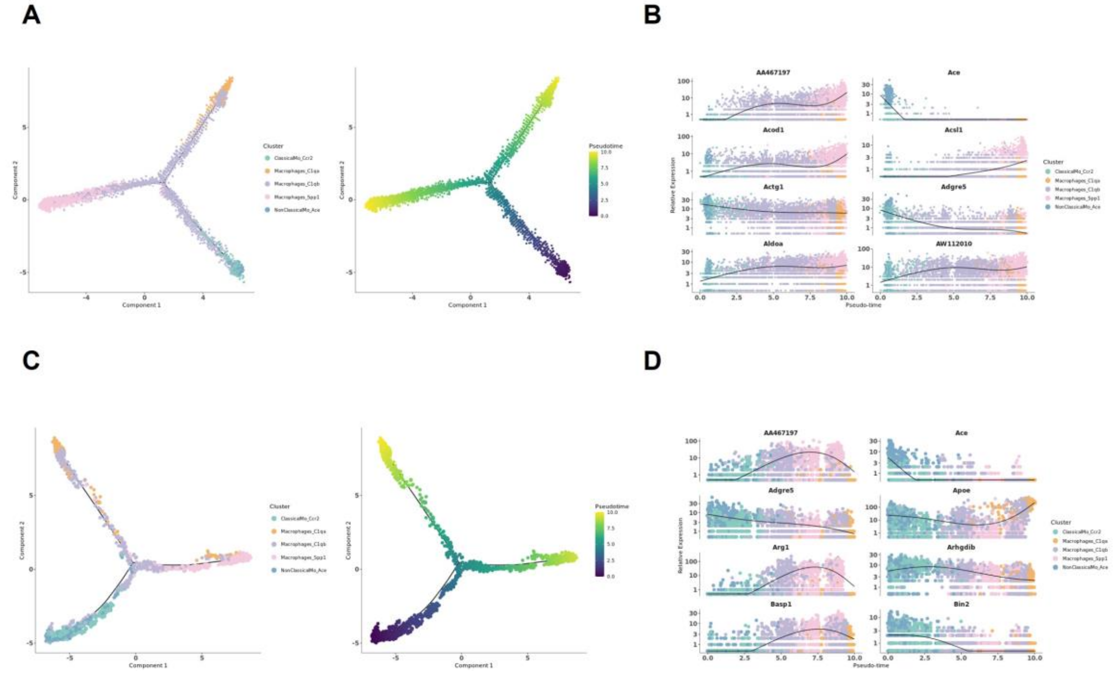


**Supplemental Figure S5.** Computational pseudotime analysis of five pulmonary monocyte–macrophage states: ClassicalMo_Ccr2, Macrophages_C1qa, Macrophages_C1qb, Macrophages_Spp1, and NonClassicalMo_Ace. (A) Cell-state and pseudotime trajectories for the 55055-infected group. (B) Selected gene-expression trends along pseudotime for the 55055-infected group. (C) Cell-state and pseudotime trajectories for the 46274-infected group. (D) Selected gene-expression trends along pseudotime for the 46274-infected group.

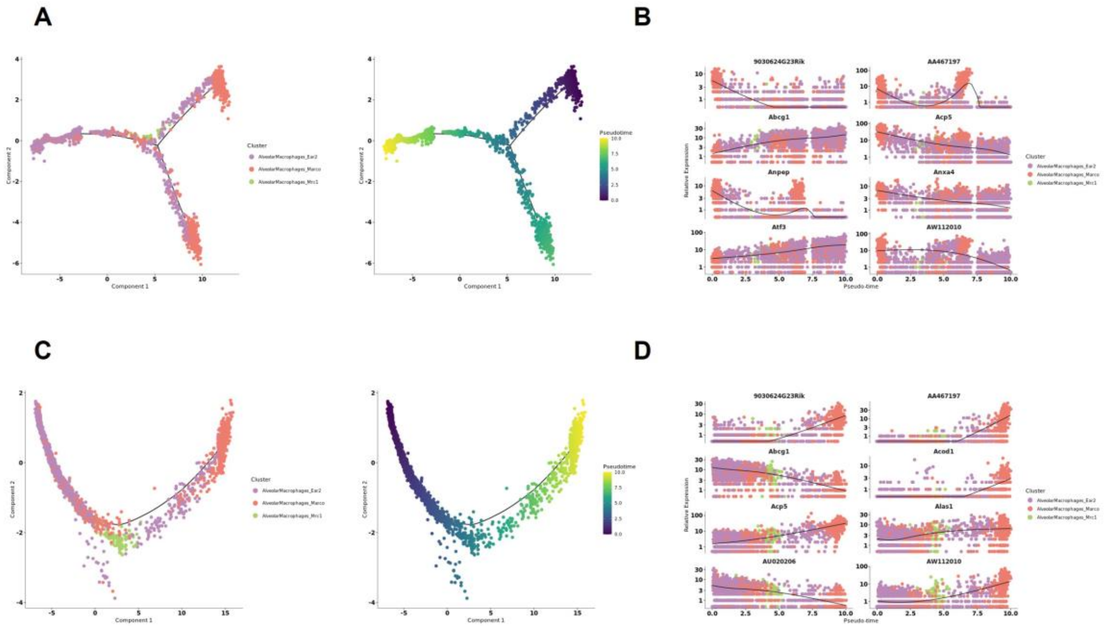


**Supplemental Figure S6.** Computational pseudotime analysis of three pulmonary alveolar-macrophage states: AlveolarMacrophages_Ear2, AlveolarMacrophages_Marco, and AlveolarMacrophages_Mrc1. (A) Cell-state and pseudotime trajectories for the 55055-infected group. (B) Selected gene-expression trends along pseudotime for the 55055-infected group. (C) Cell-state and pseudotime trajectories for the 46274-infected group. (D) Selected gene-expression trends along pseudotime for the 46274-infected group.

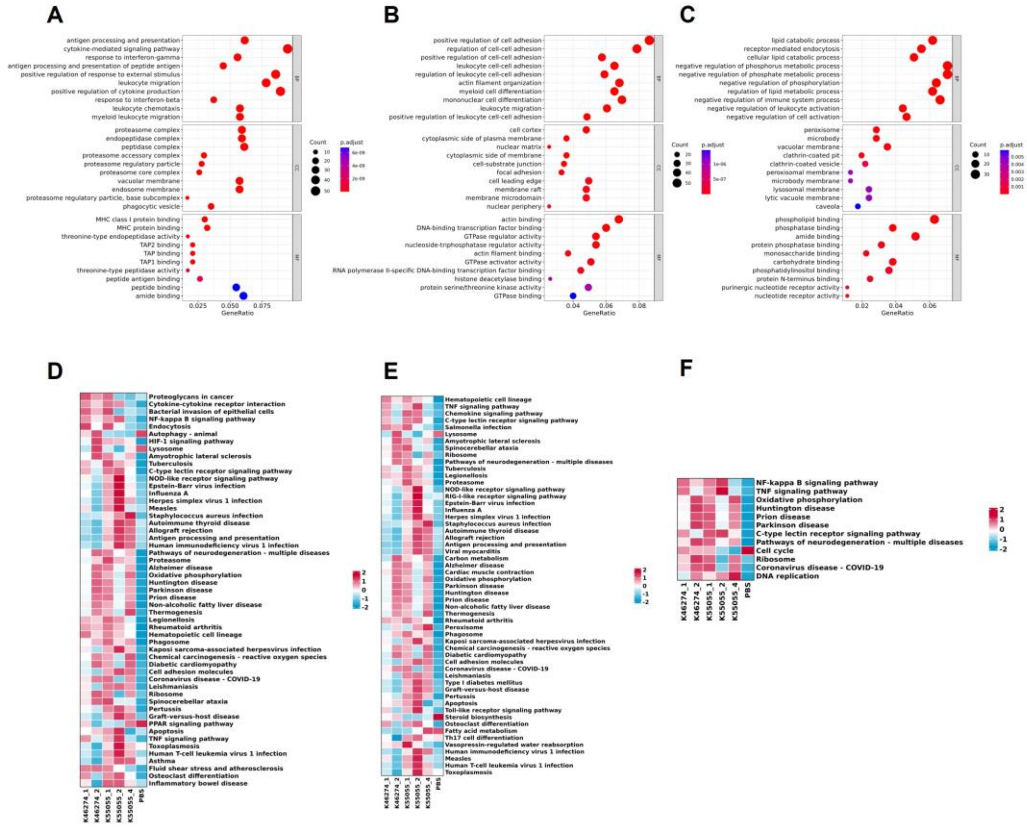


**Supplemental Figure S7.** Functional enrichment analysis of Marco⁺, Ear2⁺, and Mrc1⁺ alveolar-macrophage subsets. (A–C) Gene Ontology enrichment dot plots for the Marco⁺, Ear2⁺, and Mrc1⁺ subsets, respectively, showing biological process, cellular component, and molecular function categories. (D–F) Heatmaps of KEGG pathway scores for the corresponding subsets across pooled libraries.

**Table S1.** Primer sequences for qRT-PCR analysis

| Primer name | Primer sequence (5′–3′) |
|---|---|
| IL-6-Forward | GTACTCCAGAAGACCAGAGG |
| IL-6-Reverse | TGCTGGTGACAACCACGGCC |
| TNF-$\alpha$-Forward | TTGACCTCAGCGCTGAGTTG |
| TNF-$\alpha$-Reverse | CCTGTAGCCCACGTCGTAGC |
| iNOS-Forward | CCCTTCCGAAGTTTCTGGCAGC |
| iNOS-Reverse | GGCTGTCAGAGCCTCGTGGCTTTGG |
| β-actin-Forward | CACTGTCGAGTCGCGTCC |
| β-actin-Reverse | TCATCCATGGCGAACTGGTG |
